# mbSparse: an autoencoder-based imputation method to address sparsity in microbiome data

**DOI:** 10.1080/19490976.2025.2552347

**Published:** 2025-09-01

**Authors:** Changlu Qi, Yiting Cai, Guoyou He, Kai Qian, Mian Guo, Liang Cheng

**Affiliations:** a College of Bioinformatics Science and Technology, Harbin Medical University, Harbin, HL, China; b Department of Neurosurgery, The Second Affiliated Hospital, Harbin Medical University, Harbin, HL, China; c National Health Commission (NHC) Key Laboratory of Molecular Probes and Targeted Diagnosis and Therapy, Harbin Medical University, Harbin, China

**Keywords:** Microbiome, deep learning, sparsity, imputation

## Abstract

The involvement of gut microbiota in host physiological activities is crucial, yet the high sparsity of microbiome data, marked by numerous zeros in count matrices, presents huge analytical challenges. To overcome this, we developed mbSparse, an imputation algorithm that leverages deep learning rather than traditional predefined count distributions. Utilizing a feature autoencoder for learning sample representations and a conditional variational autoencoder (CVAE) for data reconstruction, mbSparse effectively integrates these processes to enhance imputation. Our results demonstrate that mbSparse achieves exceptional accuracy, with mean squared error reductions of up to 4.1 compared to existing microbiome methods, even amid outlier samples and varying sequencing depths. In colorectal cancer analysis, mbSparse increases the detection of validated disease-associated taxa from 7 to 27, while predictive accuracy improves, as evidenced by area under the precision-recall area under the curve values rising from 0.85 to 0.93. Additionally, mbSparse addresses non-biological zeros by restoring over 88% of removed counts and achieving a Pearson correlation of 0.9354 at a 10% removal rate, preserving essential taxonomic relationships. Finally, our exploration of mbSparse variants reveals that the CVAE is critical for enhancing accuracy, providing valuable insights for further optimizing microbiome data imputation techniques.

## Introduction

Numerous studies have highlighted the intricate relationship between the microbiota and essential physiological processes.^1–4^ Parallel to this growing body of research, the Human Microbiome Project (HMP)^5^ has been making much progress. Simultaneously, advancements in high-throughput sequencing technologies^6^ have resulted in the generation of extensive microbiome datasets. These datasets offer researchers unprecedented opportunities to perform comprehensive analyses of the microbiome and its connection to human health. As a result, the human gut microbiota is gaining recognition as a noninvasive biomarker for disease screening and diagnosis, and, in some cases, as a key target for therapeutic intervention.^7^

Regardless of the sequencing method employed,^8–11^ a challenging characteristic of the resulting taxonomic abundance matrices is the presence of numerous zero-counts, with zero-inflation reaching up to 90% in some microbiome datasets,^12^ indicating data sparsity.^13^ These zeros can be classified into three types based on their source^14^ :biological zeros, where specific taxa are genuinely absent in certain samples and do not require correction; sampling zeros, which arise from limited sequencing depth^15^ or high sequence diversity; and technical zeros, caused by biases such as DNA extraction bias,^16^ batch effects,^17,18^ and PCR bias.^19,20^ Sampling zeros and technical zeros can collectively be referred to as non-biological zeros or false zeros.

The sparsity of microbiome data poses great challenges in adequately mining and utilizing the abundance data, leading to incorrect feature identification and consequently biased research results. To address these issues, Jiang *et al*. first applied imputation strategies^21–24^ to alleviate sparsity in microbiome data and developed the mbImpute method.^25^ mbImpute employs a Gamma-normal mixture model to borrow information from analogous samples, similar taxa, and optional metadata (such as sample features and taxon phylogeny) to identify and recover potential false zero abundances. Subsequently, Zeng *et al*. introduced mbDenoise,^26^ which uses a statistical model based on zero-inflated probabilistic principal component analysis. This model is fitted using variational approximation algorithms and uses posterior mean estimation to recover the true abundance matrix, thereby achieving the goal of denoising the data. Similar to microbiome data, sparsity is also a prominent characteristic of single-cell RNA-sequencing (scRNA-seq) data for which various imputation methods, such as SAVER^27^ and the deep learning-based GE-Impute,^28^ have been designed to address this issue.

The insufficient characterization of the human microbiota, driven by its high diversity, inter-individual host variability, and technical limitations of sequencing technologies, results in extreme data sparsity that poses significant challenges for data modeling and analysis. Deep learning algorithms have shown promising efficacy in addressing such complex challenges in the data.^29^ Among these, autoencoders^30^ – a type of deep learning method – are notable for their ability to extract informative encoded features through data dimensionality reduction and reconstruction, thereby simplifying data while emphasizing critical information. Autoencoders come in various forms, including Variational Autoencoders (VAEs)^31^ and Conditional Variational Autoencoders (CVAEs),^32^ each designed to tackle challenges such as high dimensionality, sparsity, and compositional complexity in microbiome research. Various studies have applied deep learning techniques to analyze microbiome data. For instance, DeepMicro^33^ employs various autoencoders to extract pivotal disease-predictive insights from metagenomic data. CCVAE^34^ enhances data analysis accuracy by using VAEs in conjunction with assembly graphs to represent nodes sharing similar features. Additionally, methods like MetaDEC,^35^ CLMB,^36^ and VAMB^37^ utilize autoencoders for feature extraction, denoising the data, and data analysis. With their robust representation learning capabilities, deep learning techniques offer advantages in microbiome data analysis. These algorithms have shown potential in effectively addressing data complexity and sparsity, extracting essential features crucial for disease prediction and other biological applications. Thus, deep learning is poised to become a critical tool driving advancements in microbiome research.

Here, we propose mbSparse, a novel method that simultaneously applies a feature autoencoder and a CVAE for microbiome data imputation. mbSparse leverages the strengths of deep learning while introducing innovative approaches to enhance data quality. Initially, a feature autoencoder extracts key features from the samples. Subsequently, a sample correlation graph is constructed using K-nearest neighbors (KNN)^38^ to capture relationships between samples. mbSparse then employs a CVAE to generate the reconstructed data, enhancing data accuracy and completeness. By integrating insights from the sample correlation graph and the reconstructed data, mbSparse imputes zero values in the data to recover missing taxonomic unit counts. mbSparse effectively addresses sparsity in microbiome data, providing robust data support for subsequent analyses, such as identifying taxonomic units and performing differential abundance (DA) analysis. This comprehensive approach not only improves data completeness but also facilitates more accurate and reliable microbiome research.

In our experiments, we applied mbSparse to whole-genome sequencing (WGS) data, and conducted thorough comparisons and evaluations against two leading microbiome imputation methods, two advanced scRNA-seq imputation methods, and a generic imputation method (SoftImpute). Results demonstrated mbSparse’s superior performance across multiple evaluation metrics, offering a more reliable and efficient solution for microbiome data analysis. Due to its exceptional efficiency and accuracy in data processing, mbSparse offers users a precise solution for data imputation.

## Methods

### Datasets

One 16S rRNA gene sequencing dataset: We used the R package Human16sData (version 1.6.0)^39^ to obtain the human healthy stool 16S dataset which includes 187 samples and 43,140 OTUs.

Seven WGS datasets: PRJEB6070,^40^ PRJDB4176,^41^ PRJNA397219^42^ were processed and annotated to species-sample abundance matrix. PRJEB7774^43^ was obtained from the R package curatedMetagenomicData (version 3.12.0)^44^ and retrieved PRJNA398089,^45^ PRJNA615162,^46^ and PRJEB19367^47^ from the database gutMDisorder.^48^

### WGS datasets annotation

The microbial raw sequencing data were obtained from SRA or ENA. For the metagenomic datasets, quality control of raw sequencing reads was performed using FastQC.^49^ High-quality microbial reads were ensured by filtering with fastp,^50^ followed by the removal of host reads through mapping to the human genome (GRCh38/hg38) using Bowtie2.^51^ Taxonomic profiling based on reads was conducted using MetaPhlan 4.^52^

### Data definitions

(I) Complete data refers to data that does not contain any non-biological zeros. (II) Original data refers to the sparse data that is to be imputed. This is the dataset before any imputation or reconstruction has taken place. (III) Reconstructed data refers to the data generated by inputting the original data into the Conditional Variational Autoencoder. This reconstructed data is used alongside the original data for the imputation process. (IV) Imputed data is the final dataset after mbSparse has filled in the missing values, resulting in a complete dataset with no missing values.

### Preprocessing and normalization

To facilitate more accurate imputation, it is crucial to standardize the input taxonomic count matrix, ensuring uniform scaling across taxa units within each sample. mbSparse encourages users to adopt the default standardization method, which is log standardization. Users can either option for this default method or select an alternative standardization approach according to their needs. $M=\left(M_{\mathrm{ij}}\right)\in Z_{\geq 0}^{n\times m}$ is denoted as the observed count in sample *i* for taxon *j* where i=1,⋯,n and j=1,⋯,m.

To ensure uniformity in sample dimensions and align the total count of each sample with the variation library size, mbSparse conducts row-wise normalization of the counting matrix M, maintaining each sample’s total count at $10^{6}$:

(1) $$M_{ij}^{\prime }=10^{6}\cdot \frac{M_{\mathrm{ij}}}{\sum_{j{\;}^{\prime }=1}^{m}M_{\mathrm{ij}{\;}^{\prime }}}$$

Considering the potential interference from extremely large counts in M that could affect imputation accuracy, we further applied a logarithmic transformation to the normalized matrix $M^{\prime }$ to derive the final input matrix $X=\left(X_{\mathrm{ij}}\right)\in R_{\geq 0}^{n\times m}$:

(2) $$X_{\mathrm{ij}}=\mathrm{lo}g_{10}\left(M_{ij}^{\prime }+1\right)$$

### The overview of the mbSparse algorithm

To address the challenge of sparsity in microbiome data, we proposed the mbSparse algorithm, which leverages autoencoder principles. This algorithm involves three key steps: (i) constructing a sample correlation graph, (ii) employing a CVAE for data reconstruction, and (iii) imputing zero values within the data, informed by insights derived from both the sample correlation graph and the reconstructed data.

Specifically, samples that exhibit similar microbial distributions may provide valuable insights for samples undergoing imputation. To effectively integrate information from these similar samples into the target sample, we first constructed a sample correlation graph to visualize relationships among samples. Subsequently, we used this correlation graph to impute zero values within the target sample.

In order to more accurately measure the correlation between each sample, we employed a feature autoencoder to capture representative embeddings of the samples. The taxonomic count matrix is input into the feature autoencoder, which learned these representative embeddings. After the feature autoencoder was fully trained, it was used to extract representative embeddings for each sample. Subsequently, we utilized the KNN algorithm to construct a sample correlation graph based on the acquired embeddings. This process involved connecting samples with high similarity in distribution to the target sample as its neighbors. To minimize the influence of dissimilar samples, we typically set a smaller k value in the KNN algorithm. This strategic choice ensured that only information from samples with high similarity to the target sample was utilized, thereby reducing interference.

**Figure 1.**
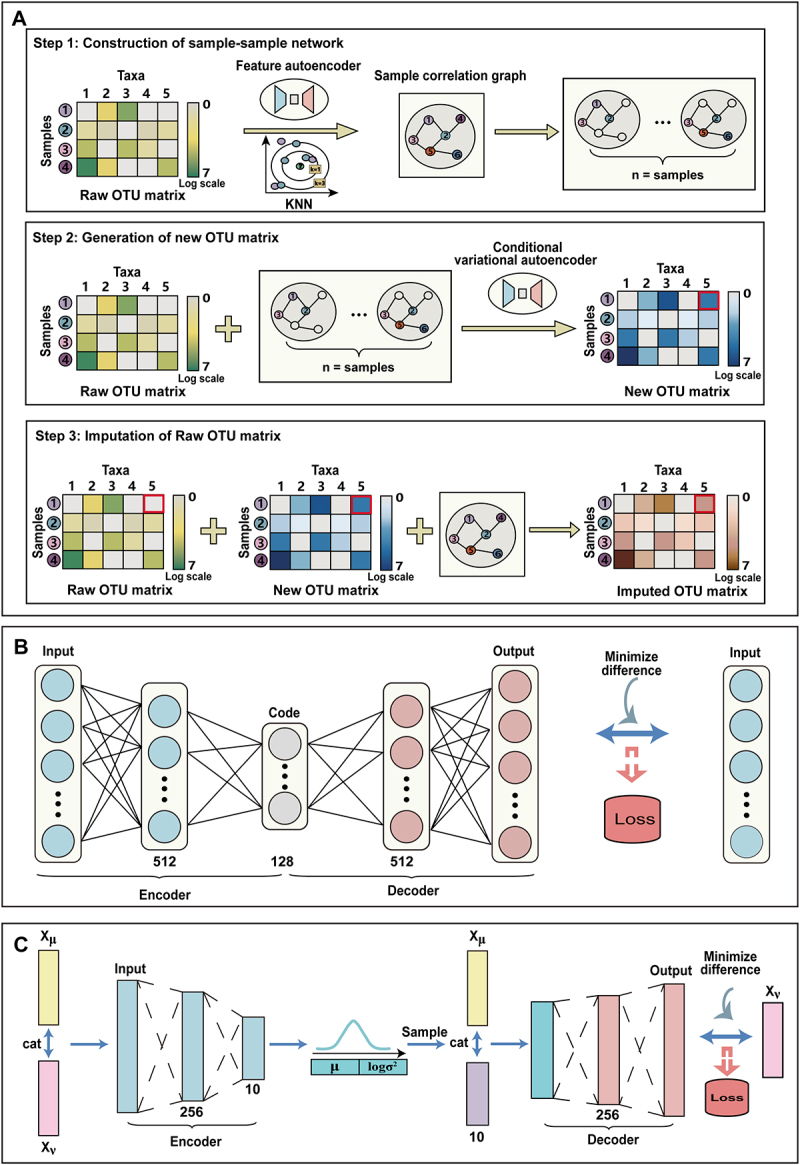
The architecture of mbSparse.

However, the number of neighboring samples around the target sample is limited, and the presence of outlier samples may greatly reduce accuracy. To enhance the reliability of information derived from neighboring samples, we employed CVAE to learn and model the relationships between the target sample and its neighbors. Subsequently, the CVAE was employed to generate conditional neighbor samples with microbial distributions similar to those of the actual neighboring samples, ensuring an equal number of conditional and actual neighbors. This step enhanced the stability of the information provided. Finally, by leveraging information from both neighboring samples and conditional neighboring samples, the zero values of the target sample were imputed, resulting in a more robust and accurate imputation process (Figure 1).

(A) Step 1, the feature autoencoder captures embeddings from the taxonomic count matrix. These embeddings are used to construct the KNN-based sample correlation graph, connecting similar samples as neighbors. Step 2, to improve accuracy, the CVAE models relationships between the target sample and its neighbors, generating conditional samples with microbial distributions that are comparable. Step 3, imputation combines information from both observed and conditional neighbors, effectively filling in zero values in the target sample. (B) The feature autoencoder processes the taxonomic count matrix as its input. It is trained by minimizing the difference between the input and output matrices. The encoder and decoder layers have dimensions of 512 × 128 and 128 × 512, respectively. (C) The CVAE takes both the taxonomic count matrix and the neighbor taxonomic count matrix as input. It is trained to maximize the evidence lower bound. The encoder and decoder layers have dimensions of 256 × 10 and 20 × 256, respectively.

### Feature autoencoder

The feature autoencoder is a pivotal technique in deep learning, responsible for transforming original data into a new feature space. This transformation enhances the representation of the data’s inherent characteristics, thereby improving the efficiency and manageability of subsequent data processing.

In our implementation, the feature autoencoder learned representative embeddings of the input matrix by employing a stack of two dense networks in both the encoder and decoder stages. Here, X was used as the input matrix for the autoencoder to obtain the representation embedding $Z\in R_{\geq 0}^{n\times d}$ where d≪m. Then, the decoder reconstructed the embedded data to obtain the reconstruction matrix $\overset{ˉ}{X}$.

During training, the autoencoder aimed to maximize the similarity between X and $\overset{ˉ}{X}$ by minimizing the target loss function, typically measured as the mean square error (MSE) between the original and reconstructed data:

(3) $$\min\overset{n}{\underset{i=1}{\sum }}\overset{m}{\underset{j=1}{\sum }}(X_{\mathrm{ij}}-{\overset{ˉ}{X}}_{\mathrm{ij}}{)}^{2}$$

This optimization process ensured that the reconstructed data closely resembled the original input, effectively capturing the essential features while minimizing information loss.

### Sample correlation graph

We define the sample correlation graph as $G=\left(V,E\right)$, where V represents the set of samples $\left\{v_{1},\cdots ,v_{n}\right\}$ where $v_{a}$ is the node corresponding to sample a and E denotes the set of edges. The adjacency matrix, denoted as $A=\left(A_{\mathrm{ab}}\right)\in \{0,1{\}}^{n\times n}$ for a≠b, assigns a value of 1 to $A_{\mathrm{ab}}$ if and only if $\left(v_{a},v_{b}\right)\in E$.

Let $N_{a}=\{v_{b}|A_{\mathrm{ab}}=1\}$ denote the neighborhood of node $v_{a}$. The sample correlation graph G leverages representative embeddings Z acquired from the feature autoencoder to quantify the relationships between samples. Enhanced similarity in representative embeddings among different samples implies a higher probability of edge existence.

To construct the graph, we employed the KNN algorithm, where k was a predefined parameter that determined the interaction scale between samples. Each node identified its *K*-nearest neighbors within a specified distance to determine the existence of edges. The similarity between samples was assessed using the Euclidean distance metric.

### Conditional variational autoencoder

CVAE is a generative model that extends the capabilities of the VAE to generate data under specific conditions. By introducing conditional variables, CVAE empowers the model to generate diverse and targeted data tailored to specified conditions.

In our approach, we utilized CVAE to learn the conditional distribution of node features for connecting neighboring samples $u\in N_{v}$ of a given sample node v, and to generate conditional samples pertaining to node v. Our CVAE construction was based on a multi-layer perceptron (MLP), incorporating two layers of MLP for encoding and decoding. We utilized $X_{v}$ as a condition, as its distribution is inherently linked to $X_{u}$.

Subsequently, we generated the latent variable z from $p_{\theta }(z|X_{v})$, and the data $X_{u}$ is generated by the generative distribution $p_{\theta }(X|X_{v},z)$ conditioned on z and $X_{v}$:$z∼p_{\theta }(z|X_{v})$,$X_{u}∼p_{\theta }(X|X_{v},z_{v})$.

Thus, we obtained:

$$\log p_{\theta }(X_{u}|X_{v})=\int q_{\phi }(z|X_{u},X_{v})\log\frac{p_{\theta }(X_{u},z|X_{v})}{q_{\phi }(z|X_{u},X_{v})}\mathrm{dz}$$

(4) $$+\mathrm{KL}(q_{\phi }(z|X_{u},X_{v})p_{\theta }(z|X_{u},X_{v}))\geq \int q_{\phi }(z|X_{u},X_{v})\log\frac{p_{\theta }(X_{u},z|X_{v})}{q_{\phi }(z|X_{u},X_{v})}\mathrm{dz}$$

where ϕ represents the variational parameters and θ denotes the generative parameters. Therefore, we obtained that the training objective of CVAE is to maximize the Evidence Lower Bound (ELBO) loss:

(5) $$\begin{array}{c} L\left(X_{u},X_{v};\theta ,\phi \right)=-\mathrm{KL}(q_{\phi }(z|X_{u},X_{v})∥p_{\theta }(z|X_{v}))\\ +\frac{1}{k}\overset{K}{\underset{i=1}{\sum }}\;\log\;p_{\theta }\left(X_{u}|X_{v},z^{\left(l\right)}\right) \end{array}$$

Where $z^{\left(l\right)}=g_{\phi }\left(X_{v},X_{u},e^{\left(l\right)}\right),e^{\left(l\right)}∼N\left(\theta ,I\right)$, k=1,…,K denotes the number of neighbors of node v. During the training phase, we feed $\left(X_{v},X_{u}\right)$ into the CVAE and train it by maximizing the ELBO loss. However, in the process of generating new conditional samples, we exclusively utilized the sample node $X_{v}$ as the input to the CVAE. Subsequently, we obtained the conditional sample $X_{v}^{c}$ associated with node v. Ultimately, for each sample, we generated its conditional samples, ensuring that the relationships between these conditional samples are consistent with sample correlation graph G. In essence, for every sample, in addition to the information from the original neighbor samples, we also incorporated information from the conditional neighbor samples. This approach enriches the information regarding neighbor samples for subsequent imputation tasks.

### Imputation strategy

We proceeded by using both the sample correlation graph and the count values of the generated conditional samples to impute the zero values in the original samples. Multiple imputation^53^ is a commonly used and effective strategy for handling missing data. It generates multiple imputed datasets and combines the estimates from each dataset to derive the final imputed value. However, in microbiome data, zero values can be either missing at random or missing not at random. Moreover, traditional multiple imputation methods do not fully account for the complex relationships between samples, which may lead to suboptimal performance in certain cases. Nevertheless, our imputation strategy draws on the ideas of multiple imputation but addresses its limitations. Specifically, we combine the reconstructed data, which captures the learned associations between samples, with the original data to impute missing values. This approach allows us to not only rely on the original data for imputation but also integrate the reconstructed data while taking into account the associations between samples, improving the accuracy of the imputation.

We applied imputation to all zero values in the data, meaning that when a value is zero, we perform imputation. However, the imputed value may still be zero. This is because we employ an imputation strategy based on similar samples; if all similar samples have a zero value in the corresponding category, the imputed result may still be zero. In this way, we can automatically identify and impute potential missing data zeros while ensuring that potential biological zero values remain unchanged, allowing for a more accurate handling of zeros in microbiome data. Specifically, for a taxon j of sample i, if its original count value $X_{\mathrm{ij}}=0$, we employ the following imputation method:

(6) $$X_{\mathrm{ij}}=\frac{1}{2K}\overset{K}{\underset{k=1}{\sum }}\left(X_{\mathrm{kj}}+X_{kj}^{c}\right)$$

where K denotes the number of neighboring samples of sample i, $X_{\mathrm{kj}}$ represents the count value of taxon j for the neighboring sample k of sample i, and $X_{kj}^{c}$ denotes the count value of taxon j for the conditional sample of sample k. If the original count value $X_{\mathrm{ij}}\neq 0$, its original value is retained to avoid interference with existing information. This imputation strategy helps in inferring meaningful values for zero count entries, leveraging information from both the original samples and their corresponding conditional samples.

## Results

### mbSparse demonstrates superior imputation performance

We assessed the imputation accuracy of mbSparse by comparing imputed data with reconstructed data, removing non-biological zero values from WGS data, which included samples from colorectal cancer (CRC), colorectal adenomas (CA), and healthy individuals. Two simulation schemes were designed for this purpose. Scheme 1 generated complete data by extracting data at varying proportions (Supplementary Experimental detail: Generation of complete data for Scheme 1), while Scheme 2 employed extraction followed by sampling to generate complete data (Supplementary Experimental detail: Generation of complete data for Scheme 2). Each scheme introduced non-biological zeros through drop-out experiments (Supplementary Experimental detail: Dropout step), which were then imputed using different methods.

Given the limited number of imputation methods for microbiome data – specifically, mbImpute and the mbDenoise variants (mbDenoise-lmn and mbDenoise-nb) – we introduced two additional methods suitable for scRNA-seq data (SAVER and GE-Impute), as well as a general method SoftImpute (Supplementary methods: Imputation methods). To numerically compare the imputation accuracy of these different methods, we used three indicators: the mean squared error (MSE) between the imputed data and the complete data, and the Pearson correlation between imputed abundances and complete abundances for each taxon, and the Spearman correlation between imputed and complete abundances for each taxon. Differences observed across different data types for the two mbDenoise variants indicated that these methods heavily depended on model selection and lacked adaptability across diverse data types.

**Figure 2.**
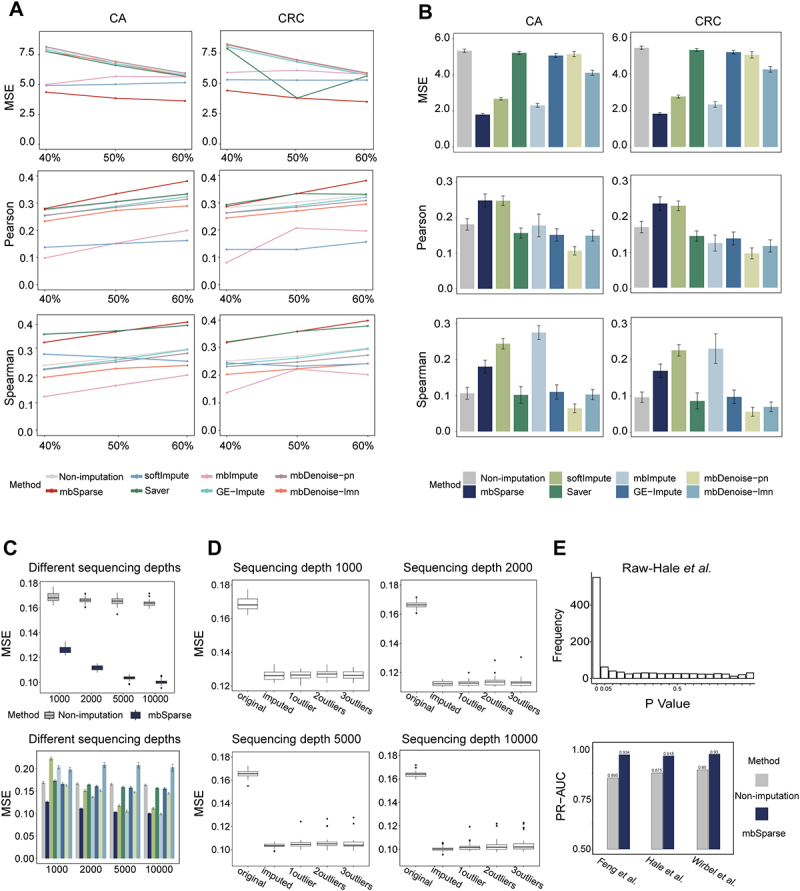
mbSparse imputation performance evaluation and robustness analysis.

mbSparse, tested with various percentage settings in Scheme 1, consistently outperformed other models in most cases across all three evaluation indicators. Following imputation via mbSparse, MSE values decreased by nearly half compared to non-imputed data across all percentage settings. When compared with scRNA-seq imputation methods, MSE value reductions ranged from a minimum of 2.10 to a maximum of 3.89. In comparison with existing microbiome imputation methods, MSE value reductions ranged from 0.82 to 4.14 (Figure 2(A)). Similarly, Pearson correlation exhibited a comparable trend. Moreover, Spearman correlation was slightly lower than SAVER in only 40% of cases but outperformed other methods in all remaining scenarios (Figure 2(A)). In Scheme 2, all three evaluation indicators showed performance similar to that observed in Scheme 1. Although the Spearman correlation was not the highest, it still outperformed most other methods (Figure 2(B)), affirming mbSparse’s advantages over current microbiome and some scRNA-seq imputation methods.

(A) The x-axis represents different extraction ratios, while the y-axis shows three evaluation metrics: MSE, Pearson correlation coefficient and Spearman correlation coefficient. Line plots illustrate the imputation performance of mbSparse compared to other methods (softImpute, Saver, GE-Impute, mbImpute, and mbDenoise), with no imputation serving as the baseline. (B) The x-axis indicates various imputation methods. Bar charts, based on the results of 30 repeated experiments, demonstrate the overall performance advantage of mbSparse over other methods, using no imputation as the baseline. (C) The robustness of mbSparse at different sequencing depths is assessed using MSE. The top panel displays box plots that evaluate mbSparse’s imputation performance across four sequencing depths (1000, 2000, 5000, and 10,000 reads) compared to no imputation, based on 30 repeated experiments. The bottom panel features bar charts that compare the performance of mbSparse and other methods at different sequencing depths, with each method represented by different colors consistent with those in panel. (D) Box plots are created to evaluate mbSparse’s performance with datasets containing varying numbers of outlier samples, across different sequencing depths, based on 30 repeated experiments. Imputed results are used as the baseline. (E) The top panel presents a histogram showing the p-value distribution from differential analysis performed using edgeR on the raw Hale *et al*. dataset. The bottom panel features a bar chart illustrating the classification accuracy of an elastic net-regularized logistic regression algorithm in predicting disease status across three CRC datasets. This accuracy is measured by the area under the PR-AUC values obtained through five-fold cross-validation. Features identified by edgeR from both non-imputed (light color) and mbSparse-imputed (dark color) data are used. These features are selected based on an FDR threshold of 0.05 and an absolute log fold change (logFC) greater than 1.

The robustness of the mbSparse model was assessed from two perspectives. Firstly, its performance across varying sequencing depths was evaluated using simulated 16S rRNA data based on real microbiome data for 300 taxa in 54 healthy human fecal samples from the HMP16SData. Each sample underwent sequencing at four depths (1000, 2000, 5000, and 10,000 reads) with 30 replicates per depth. A marked enhancement in mbSparse’s imputation accuracy was observed with increasing sequencing depth (Figure 2(C)). Greater sequencing depths resulted in fewer missing data, enabling more effective utilization of non-missing data for mbSparse model training. Additionally, the same sequencing depths and repetitions were applied to five alternative imputation methods to compare and verify mbSparse’s superior robustness. Although other methods also showed improved performance with increased sequencing depth, mbSparse consistently outperformed them across all depths (Figure 2(C)).

Secondly, the model’s ability to handle outlier samples was also investigated. Using the same strategy, outlier samples were introduced into the 16S rRNA data at the four aforementioned sequencing depths. From a pool of 64 samples, one sample is randomly selected and transformed into an outlier. In this transformation, 62 low-abundance taxa, defined as those with mean abundances below the median and at least 10 non-zero abundances, are assigned random high-abundance values. These high-abundance values are sampled from the top 100 maximum abundances for each taxon. The abundances of the remaining taxa are set to zero. This created an outlier sample, allowing us to assess the model’s robustness in such scenarios. Furthermore, the impact of different numbers of outlier samples was explored by introducing two and three outlier samples in the same manner, with each scenario replicated 30 times. Across all sequencing depths, mbSparse demonstrated consistent performance. Although the presence of outlier samples caused a slight decrease in imputation accuracy, this decrease is minimal, regardless of whether one or multiple outlier samples were present (Figure 2(D)). Thus, increasing the number of outlier samples did not impair performance.

To further investigate the tolerance of mbSparse to outlier samples, we conducted additional experiments by introducing varying percentages of outlier samples relative to the original sample size. We observed that, generally, imputation performance declined as the proportion of outlier samples increased. However, in some cases, performance actually improved with a higher percentage of outlier samples (Suplementary Figure S1). This could be due to increased random connectivity among outlier samples, which reduced their impact on the original data. Despite the overall decrease in performance with more outlier samples, mbSparse consistently outperformed the no-imputation scenario. This indicated that mbSparse remains effective even when handling a substantial proportion of outlier samples. In conclusion, mbSparse exhibited robust imputation accuracy even when handling outlier samples.

### mbSparse enhances precision in identifying differentially abundant microbiota

Identifying DA taxa, which are biologically meaningful because they usually are associated with disease, is a common goal in microbiome research. In this study, DA analysis is employed on three CRC datasets using five methods (Linear discriminant analysis Effect Size (LEfSe) analysis,^54^ Wilcoxon rank-sum test, DESeq2_phyloseq,^13,55^ edgeR,^56^ and ALDEx2^57^) to detect DA taxa in both non-imputed and mbSparse-imputed data (Supplementary methods: DA analysis methods). Our model relied solely on sample–sample similarity, avoiding the inclusion of sample grouping information to prevent inflated within-group similarity and model overfitting. All five methods successfully identified DA taxa in each dataset, regardless of whether imputation was used as a preprocessing step. To comprehensively evaluate the analysis outcomes, we plotted the distribution of p-values before and after imputation for each dataset separately (Figure 2(E); Supplementary Figure S2-S6). Our goal was to ascertain whether the distribution conformed to expectations, particularly whether a majority of p-values concentrated in the 0–0.05 range, with a uniform distribution elsewhere. Notably, only edgeR met this expectation regardless of imputation, prompting us to prioritize edgeR results. The identified DA taxa from non-imputed and mbSparse-imputed data were used as features in an elastic net-regularized logistic regression algorithm to predict phenotype. The predictive accuracy was evaluated using the precision-recall area under the curve (PR-AUC) through 5-fold cross-validation (Supplementary Experimental detail: classification). These PR-AUC values for all mbSparse-imputed data were higher compared to the corresponding non-imputed datasets, indicating that the imputation method enhanced the accuracy of distinguishing DA taxa and improved disease classification performance (Figure 2(E)). The Feng *et al*. dataset showed the most substantial performance increase, with PR-AUC rising from 0.85 to 0.93, prompting us to concentrate subsequent analyses on this dataset. We compared the DA taxa identified before and after imputation that contributed to distinguishing phenotypes. We compared the DA taxa identified before and after imputation that contributed to distinguishing phenotypes. Specifically, when using elastic net-regularized logistic regression for phenotype classification, the algorithm assigned weights to each taxon to assess its importance in the classification, thereby measuring how each taxon contributed to differentiating between phenotypes. These were, respectively, compared against taxa annotated with CRC functional terms in the GMrepo database.^58^ The number of taxa with a weight greater than zero increased from 29 in the non-imputed data to 55 in the mbSparse-imputed data. Moreover, the number of taxa annotated with CRC functional terms in GMrepo increased from 7 (non-imputed) to 27 (mbSparse-imputed).

We collected and organized existing literature on CRC-associated taxa verified as significantly altered in individuals with CRC.^59–65^ Surprisingly, in this dataset, none of the non-imputed taxa with a weight greater than zero were validated in the literature. However, mbSparse-imputed, several taxa are validated as significantly altered in individuals with CRC, including *Gemella haemolysans*, *Bifidobacterium longum*, *Erysipelatoclostridium ramosum*, *Eubacterium rectale* and *Bacteroides uniformis*, while *Faecalibacterium prausnitzii* and *Eubacterium siraeum* are significantly decreased. Specifically, *Bifidobacterium longum* has been shown to suppress CRC by modulating gut microbiota and immune function, as well as through the regulation of oncogenes and tumor suppressor miRNAs in mouse models.^66,67^ Additionally, *Eubacterium rectale* has been implicated in CRC development through the promotion of colitis.^68^ These findings suggested that mbSparse was more effective in identifying CRC-associated taxa, providing a valuable basis for further analysis.

Furthermore, we conducted intersecting analyses on the DA taxa identified before and after imputation across the three CRC datasets. Specifically, EdgeR identified 3 DA taxa, whereas mbSparse-imputed edgeR identified 11 DA taxa in CRC samples compared to healthy samples. Notably, *Faecalicoccus pleomorphus*, which was identified in the analysis using only the mbSparse-imputed data, is significantly decreased in CRC compared to healthy samples according to the literature.^61,64^ Overlap analysis of CRC datasets demonstrates that mbSparse aided in recovering DA taxa detected in one dataset but undetected when imputation is not used. This highlighted mbSparse’s effectiveness in improving the detection and recovery of biologically significant taxa in microbiome research.

To further investigate whether mbSparse could enhance the biological significance of DA taxa identified by edgeR, identification of DA taxa was validated by using functional terms from the GMrepo database. Specifically, we focused on a CRC-related term: “Tumors or cancer of the COLON or the RECTUM or both; Risk factors for colorectal cancer include chronic ULCERATIVE COLITIS; FAMILIAL POLYPOSIS COLI; exposure to ASBESTOS; and irradiation of the CERVIX UTERI.” Using Fisher’s exact test, we compared the union identified DA taxa against those annotated under this term in GMrepo. After applying mbSparse, the p-value from Fisher’s test decreased from 0.3653 to 3.587e-8, indicating a stronger enrichment of the identified DA taxa (Supplementary Table S1-S2). This confirmed that mbSparse enhanced edgeR’s ability to identify biologically relevant DA taxa.

In addition to evaluating the model’s performance on WGS data, we further validated its imputation ability on 16S rRNA sequencing data. To achieve this, we used the 16S simulator sparseDOSSA to generate the abundances of 200 taxa in 100 samples under two conditions. Among these 200 taxa, 50 were predefined as truly DA taxa. To comprehensively assess the performance of mbSparse on 16S rRNA sequencing data, we applied five DA methods: LEfSe, Wilcoxon, DESeq2-phyloseq, edgeR, and ANCOM. By comparing the DA taxa detected by each method with the truly DA taxa, we calculated precision, recall, and F1 scores to evaluate the accuracy of DA identification. Across different DA methods, mbSparse consistently outperforms the non-imputed data in terms of precision, recall, and F1 scores (Supplementary Figures S8–S12), confirming the effectiveness of mbSparse imputation. Notably, edgeR and DESeq2 yield precision, recall, and F1 scores of zero on the non-imputed data. This phenomenon may be attributed to the fact that both methods are based on the negative binomial distribution assumption, whereas the simulated data were generated using a zero-inflated log-normal distribution. This mismatch between model assumptions and data distribution likely hinders the effective identification of DA taxa. However, after imputation with mbSparse, edgeR and DESeq2 successfully identified some DA taxa, indicating that the imputation process improved the data distribution characteristics and enhanced the applicability of these DA methods.

### mbSparse enhances detection of non-biological zero

In practical applications, non-biological zeros resulting from technical biases need to be identified and corrected to enhance the overall quality of datasets, ensuring the accuracy of subsequent analytical results. To validate mbSparse’s capability in handling non-biological zeros, we used Feng *et al*. where non-zero counts were systematically removed at varying rates to simulate non-biological zeros. mbSparse was then employed to impute and restore these removed counts, with the experimental procedures repeated independently 30 times for consistency. mbSparse successfully identified over 88% of newly introduced non-biological zeros across all removal rates (Table 1; Supplementary Table S3-S4). The effectiveness in imputing non-biological zero counts was assessed through Pearson correlation coefficients and Spearman correlation coefficients, comparing correlations between the count matrix after removal and the original matrices, both before and after mbSparse application. Increases in Pearson correlation was observed using mbSparse-imputed data, notably achieving a correlation of 0.9354 at a 10% removal rate. Even at higher removal rates (up to 70%), which introduced numerous non-biological zeros, substantial correlation enhancements were evident, affirming mbSparse’s efficacy in restoring non-biological zeros. Similarly, the application of mbSparse led to an improvement in Spearman correlation results. While the Spearman correlation for mbSparse-imputed data was slightly lower than that of the non-imputed data at the 10% and 20% removal rates, it consistently outperformed the non-imputed data at higher removal rates. These results further highlight the effectiveness of mbSparse in restoring non-biological zeros.

**Table 1.** Evaluation of mbSparse in identifying zeros due to downsampling of Feng *et al*.‘s CRC WGS dataset. For each removal rate of 10% and 20%, the first row lists the average percentage of downsampling zeros identified by mbSparse; the second row lists the average Pearson correlation between a downsampled matrix and the original matrix (on the log-scale) before imputation; the third row lists the average Pearson correlation (on the log-scale) after mbSparse is used; the fourth row lists the average Spearman correlation between a downsampled matrix and the original matrix (on the log-scale) before imputation; the fifth row lists the average Spearman correlation (on the log-scale) after mbSparse is used. Each average is calculated across 30 downsampling replicates, with the standard deviation provided as the margin of error.

Removal rate	10%	20%
% of downsampling zeros identified	90.10%±1.06%	88.17%±1.48%
Pearson correlation non-imputed	0.4737 ± 1.5003e-06	0.4735 ± 8.0812e-06
Pearson correlation mbSparse-imputed	0.9354 ± 0.0024	0.8957 ± 0.0034
Spearman correlation non-imputed	0.9550 ± 5.393711e-05	0.9074 ± 0.0001
Spearman correlation mbSparse-imputed	0.9261 ± 0.0046	0.9014 ± 0.0060

**Figure 3.**
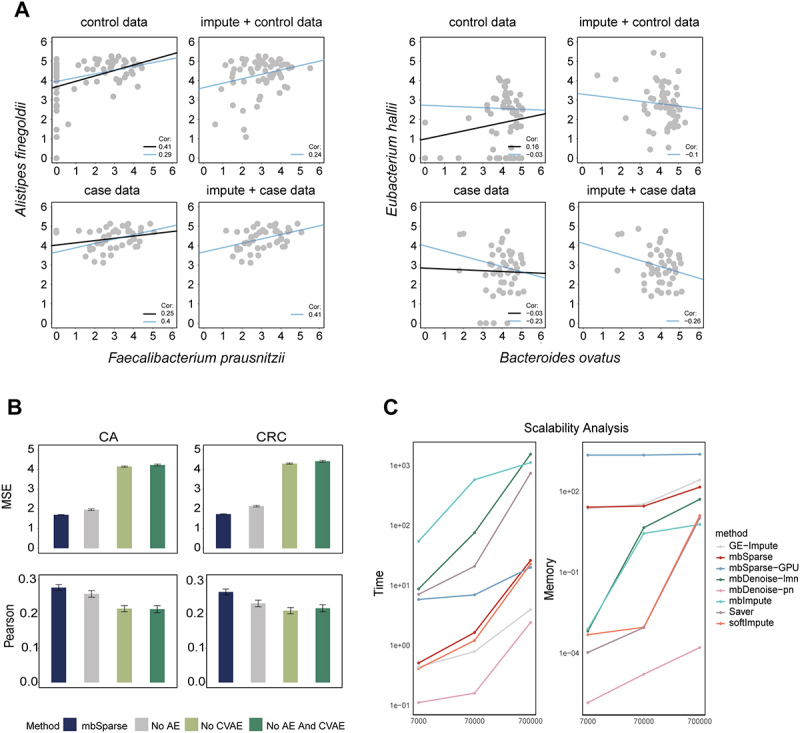
mbSparse’s ability to retain non-zero abundance distribution of taxa and ablation analysis.

To further validate mbSparse’s ability to maintain non-zero abundance features, we analyzed pairwise taxonomic relationships studied by Feng *et al*. Pearson correlations were calculated of original data on the log scale for three randomly selected taxonomic pairs. Differences in Pearson correlation were initially observed between all samples and non-zero samples in both control and diseased groups. After applying mbSparse, correlations among all imputed samples more closely mirrored those among the original non-zero samples, effectively reducing disparity and preserving the distribution characteristics of non-zero samples. Additionally, linear relationships among these taxonomic pairs were also explored using standard major axis regression (SMA). Differences in regression line slopes were observed between all samples and non-zero samples in the original data, sometimes displaying opposite trends. Following mbSparse application, the slopes of regression lines for all samples became more analogous to those observed in non-zero samples from the original data (Figure 3(A)).

(A) mbSparse preserves the distribution characteristics of non-zero abundances. Four scatter plots illustrate the relationships between microbial abundances, analyzing data according to phenotype with and without the use of mbSparse as a preprocessing step. The left panels show two SMA regression lines based on the non-imputed data and their corresponding Pearson correlations (black: based on all samples; blue: based on samples where both taxa have non-zero abundances). The right panels display the SMA regression line (blue) and Pearson correlation using all samples from the mbSparse-imputed data. (B) The impact of different mbSparse components on imputation performance is evaluated. Three variants are designed: one without the feature autoencoder, one without the CVAE, and one without both autoencoders. Bar charts are used to assess and compare the performance of these variants. (C) An analysis of runtime and memory consumption to evaluate the scalability of different methods on large-scale datasets. The x-axis represents the dataset size (samples × taxa), while the y-axes of the left and right subplots represent runtime (in seconds) and memory usage (in MB), respectively. The y-axis values are log-transformed to better visualize the differences in runtime and memory usage across varying dataset sizes.

To further investigate mbSparse’s capability in preserving inherent relationships within the data, we conducted a literature search on the DA taxa identified by edgeR only after imputation. Specifically, *Alistipes finegoldii* and *Faecalibacterium prausnitzii* showed significant reductions in CRC, whereas *Gemella haemolysans* and *Bacteroides stercoris* exhibited significant increases. Three pairs of these taxa were selected for comprehensive analysis, *Alistipes finegoldii-Faecalibacterium prausnitzii*, *Alistipes finegoldii-Gemella haemolysans*, and *Gemella haemolysans-Bacteroides stercoris*, covering scenarios of decrease–decrease, decrease–increase, and increase–increase. Following similar procedures, Pearson correlations and regression slopes were examined for these pairs. Prior to imputation, discrepancies in both correlations and slopes were initially noted between all samples and non-zero samples in the original data. After implementing mbSparse, these differences were substantially minimized, aligning the imputed abundances more closely with original non-zero values (Figure 3(A); Supplementary Figure S7). These taxa, which were identified exclusively after imputation, could be attributed to the masking effect of prevalent zero values on the abundance differences prior to imputation, which hindered their detection by edgeR. mbSparse effectively resolved this issue. These findings further validate mbSparse’s efficacy in preserving characteristic relationships within the data and underscore its significance in enhancing microbiome data analysis.

### Assessing the impact of mbSparse components on imputation performance

Building on the need to optimize imputation techniques for microbiome data, we designed three variants of the mbSparse method to explore the influence of its individual components on overall performance. The first variant omitted the feature autoencoder, using the original features directly as input. The second variant excluded the CVAE, relying solely on sample similarity graphs for imputation. The third variant involved removing both the feature autoencoder and the CVAE. Comparing the performance of these variants with the full mbSparse method provided insights into the contribution of each component to imputation effectiveness.

Experiments were conducted on two datasets generated by Scheme 2. Removing the CVAE led to the most substantial decline in imputation performance, followed by the removal of the feature autoencoder (Figure 3(B)). This highlights the crucial role of the CVAE in imputing microbiome data, as its absence markedly impairs imputation performance. The presence of the CVAE effectively learns the distribution of sample data and generates new samples with certain statistical characteristics, enhancing imputation accuracy. In contrast, while the removal of the feature autoencoder also reduces performance, its impact is relatively minor, indicating that the feature autoencoder, though important for extracting original features, is less critical to imputation performance than the CVAE.

Overall, a thorough analysis of the different components of the mbSparse method showcases their roles and how they impact the experimental results. These findings provide valuable guidance and insights for further optimizing the mbSparse imputation method, enhancing its application in the processing and analysis of microbiome data.

### Analysis of computational efficiency and memory usage in mbSparse

Since our method is based on neural networks and can be accelerated using GPU, the evaluation was conducted in two configurations: mbSparse (CPU-only) and mbSparse-GPU (GPU-accelerated). All methods exhibit an increase in runtime as the dataset size grows, with particularly steep escalations observed in mbImpute, mbDenoise-lmn, and Saver. In contrast, the runtime of both mbSparse and mbSparse-GPU, which is relatively close to the optimal methods, increases more gradually as the dataset size expands. Notably, while mbSparse-GPU demonstrates performance comparable to the CPU-only version on smaller datasets (7,000–70,000 samples), it delivers substantially faster processing speeds on larger datasets (700,000 samples), showing the superior efficiency of GPU acceleration for large-scale data.

However, the use of GPU acceleration introduces additional memory overhead. Specifically, mbSparse-GPU incurs higher memory consumption on larger datasets, primarily due to the additional memory required for GPU processing and data transfer. In contrast, the memory consumption of most other methods increases more gradually as the dataset size grows. mbSparse (CPU-only) consumes slightly more memory than other methods, but its overall memory usage remains within an acceptable range, without compromising its scalability.

In conclusion, mbSparse achieves a good balance between time efficiency and memory consumption across varying data scales. For large datasets, the GPU-accelerated version enables runtime reduction, albeit at the cost of increased memory consumption. This “memory-for-time” trade-off is particularly advantageous in environments with sufficient computational resources and demanding processing speed requirements.

## Discussion

Sparsity is a challenging characteristic of microbiome data that necessitates effective imputation methods to ensure the accuracy and reliability of subsequent analyses. To address this challenge, we proposed the mbSparse algorithm, which leveraged autoencoders. mbSparse employed a feature autoencoder to learn sample feature representations and then constructed a sample correlation graph based on these features. The CVAE was used to reconstruct the data. Zero values were imputed using insights from the sample correlation graph and the reconstructed data, effectively mitigating sparsity.

mbSparse demonstrated good performance in reducing sparsity in microbiome data. The model’s imputation accuracy was validated by comparing imputed data with complete data across various simulation scenarios, consistently outperforming other models. Additionally, mbSparse enhanced the accuracy and reliability of differential abundance analysis methods, confirming the biological functionality of the identified taxa and reinforcing the model’s usefulness in biological contexts. Robustness was further validated by assessing the model’s performance at different sequencing depths and its handling of outliers. Furthermore, mbSparse effectively identified non-biological zeros and maintains non-zero abundance features, underscoring its importance in sparsity research.

Given that the distribution of microbiome data is not explicitly defined, imposing predefined distributions may limit the model’s applicability and bias the imputation results. Therefore, the mbSparse model does not assume any underlying distribution. When validating the two variants of the mbDenoise method (mbDenoise-lmn and mbDenoise-nb), which are based on logistic normal multinomial and zero-inflated negative binomial distributions, respectively, we observed varied results. Additionally, unexpected negative improvements in mbImpute performance at sequencing depths of 1000 and 2000 suggest a dependency on data distribution, indicating that predefined distribution assumptions might not be necessary. These findings highlight the importance of a flexible approach, as employed by mbSparse, to better accommodate the intrinsic variability of microbiome data.

Considering these observations, leveraging deep learning algorithms in model construction without predefined assumptions allows for the automatic capture of complex semantic and syntactic relationships within the data. Future research should focus on exploring various deep learning models, optimizing these models, and refining the underlying algorithms to develop approaches better suited for microbiome data.

For imputation, the model utilizes similar samples identified through the KNN algorithm, which can be determined using various methods, including phylogenetic tree information. Incorporating phylogenetic relationships, a crucial aspect of microbiome research, enhances the imputation process by providing prior knowledge and deeper insights into the data. However, it is essential to consider model fitting and the practical implications of phylogeny inferred from molecular sequences. Quantifying the uncertainty of phylogenetic inference and its impact on downstream analyses is necessary. While mbSparse shows advantages in addressing microbiome data sparsity, its generalizability across different datasets requires further validation. Future work should explore the model’s applicability in various sampling sites, such as the oral cavity, and different ecosystems like marine and soil environments, to understand its potential across diverse conditions. Additionally, the current mbSparse method is primarily designed for cross-sectional data, assuming that all samples are collected at the same time point, and therefore does not fully account for the impact of the time dimension on inter-sample relationships. Longitudinal microbiome data typically include information from multiple time points, and there may be dependencies and dynamic changes between samples. These characteristics need to be effectively captured through time series modeling. The existing model does not sufficiently consider the dynamic changes in time series, which may limit the accuracy of imputation results when handling longitudinal data.

## Supplementary Material

Supplementary_data.zip

## Code availability

The code generated by this article is available at https://github.com/liangcheng-hrbmu/mbSparse.
