# Supplementary material for: mbSparse: an autoencoder-based imputation method to address sparsity in microbiome data
Source: Gut Microbes. 2025 Sep 1;17(1):2552347. doi: 10.1080/19490976.2025.2552347 (PMC12407639; doi:10.1080/19490976.2025.2552347)
Supplement: Supplementary_data.zip [file KGMI_A_2552347_SM8816.zip › SUPPLEMENTARY DATA-2.docx]

**Supplementary for “mbSparse：an autoencoder-based imputation method to address sparsity in microbiome data”**

**Supplementary methods**

**Imputation methods**

We compared mbSparse and five widely used imputation methods. Two of these methods, namely mbImpute and mbDenoise, were designed to handle microbiome data, while SAVER and GE-Impute were designed for single-cell RNA sequencing (scRNA-seq) data. Lastly, softImpute, can handle both data types above simultaneously. Each of these methods estimate the imputed count matrix given the non-imputed count matrix. The dimensions of both matrices are the same.

**1. mbImpute**

Using ***mbImpute*** (R package, version 0.1.0), we typed in the following command in Rstudio to impute the taxonomic count matrix:

*mbImpute(otu_tab = t(taxonomic count matrix))*

1. **mbDenoise**

Using ***mbDenoise*** (R package, version 1.0.0), we typed in the following command in Rstudio to impute the taxonomic count matrix:

*ZIPPCApn(t(taxon_count_matrix), V = NULL, family = "negative.binomial", n.factors = 2, rank = FALSE, trace = FALSE, maxit = 100, parallel = TRUE)*

*ZIPPCAlnm(t(taxon_count_matrix),V = NULL, n.factors = 2, rank = FALSE, trace = FALSE, maxit = 100, parallel = TRUE)*

where ZIPPCApn is for mbDenoise-nb, while ZIPPCAlnm works for mbDenoise-lmn.

1. **SAVER**

Using ***SAVER*** (R package, version 1.1.2), we typed in the following command in Rstudio to impute the taxonomic count matrix:

*saver(taxon_count_matrix, ncores = 1, estimates.only = TRUE)*

1. **softImpute**

Using ***softImpute*** (R package, version 1.4.1), we typed in the following command in Rstudio to impute the taxonomic count matrix:

*complete(t(taxon_count_matrix), softImpute(t(data), rank.max = 10, lambda = 0, trace.it = TRUE))*

1. **GE-Impute**

Using ***GE-Impute*** (Python package, version 1.0.0), we typed in the following command in Pycharm to impute the taxonomic count matrix:

*graph_adj=Impute.GraphBuild(input_file=taxon_count_matrix,k=3)*

*cell_emb=Impute.trainCellEmbeddings(graph_adj)*

*data_imp=Impute.imputation(scfile=taxon_count_matrix,embeddingfile=cell_emb,AdjGraph=graph_adj)*

**DA analysis methods**

1. **LEfSe**

Based on the software, ***LEfSe*** (version 1.1.2), we used the following command to obtain the result of LEfSe:

*lefse_format_input.py count_input.txt count_input.in -c 2 -u 1 -o 1000000*

*lefse_run.py count_input.in count_input.res -a 1 -w 1 -l 0*

1. **Wilcoxon**

We used R package ***stats*** (version 4.2.1) and the following command to obtain the result of Wilcoxon:

*wilcox.test(x = taxon_count_matrix, paired= F)*

1. **DESeq2-phyloseq**

We used ***DESeq2*** (R package, version 1.38.3) package combined with ***phyloseq*** (R package, version 1.42.0) and the following command to obtain the result of DESeq2 :

*DESeq(phyloseq_to_deseq2(physeq2, ∼ condition), sfType = "poscounts")*

1. **edgeR**

We used R package ***stats*** (version 3.42.4) and the following command to obtain the result of edgeR:

*topTags(glmLRT(glmFit(dge, design, robust=TRUE)), n=nrow(taxon_count_matrix))*

1. **ALDEx2**

We used R package ***ALDEx2*** (version 1.30.1) and the following command to obtain the result of ALDEx2:

*aldex(taxon_count_matrix, condition, mc.samples=128, test="t", effect=TRUE,include.sample.summary=FALSE, denom="all", verbose=FALSE)*

**Supplementary Experimental detail**

**Generation of complete data for Scheme 1**

To generate the complete data for Scheme 1, we first processed the raw data according to the preprocessing and normalization steps described in the Methods section and taxon with zero counts in more than 95% of the abundance matrix were then excluded. The resulting matrix is denoted as $\boldsymbol{X}=(X_{ij})\in\mathbb{R}^{n\times m}$, where $n$ represents the number of samples and $m$ represents the number of taxa. Additionally, the covariate matrix $\boldsymbol{D=}D_{il}\in\mathbb{R}^{n\times q}$ was constructed in addition to the abundance matrix where $D_{il}$ gives the observed value of the $l^{th}$ covariate in sample $i$ for $l=1,\ldots,q$. In our experiments, based on metadata derived from the raw data, the covariates Age, Sex, and AvgSpotLen were employed. Age and AvgSpotLen were treated as continuous variables, while Sex was represented as a binary covariate. Consequently, we set $q = 3$.

Next, the set $\boldsymbol{\Omega}=\{(i,j):i=1,\ldots,n;j=1,\ldots,m\}$ was defined to include those (sample, taxon) pairs where the abundance was unlikely to be missing, meaning that these data do not contain non-biological zeros and are therefore considered complete data. To construct the set $\boldsymbol{\Omega}$, the strategy outlined in the mbImpute literature was followed. Specifically, it was assumed that the abundance of each taxon conformed to a mixture distribution model. This model comprised two components: one used a gamma distribution to model potential non-biological zeros and low abundances, while the other employed a normal distribution to model actual abundances. The mean of the normal distribution incorporated metadata information for the samples. More specifically, the abundance $X_{ij}$ of taxon $j$ in sample $i$ was assumed to follow the following mixture distribution:

$X_{ij}\sim p_{j}\cdot\Gamma(\alpha_{j},\beta_{j})+(1-p_{j})\cdot\mathcal{N(}D_{i}^{\top}\gamma_{j},\sigma_{j}^{2})$ (1)

In this model, $p_{j}\in(0,1)$ represents the probability that taxon $j$ is not detected; $\Gamma(\alpha_{j},\beta_{j})$ denotes a gamma distribution with shape parameter $\alpha_{j}>0$ and rate parameter $\beta_{j}>0$; and $\mathcal{N(}D_{i}^{\top}\gamma_{j},\sigma_{j}^{2})$ represents a normal distribution with mean $D_{i}^{\top}\gamma_{j}$ and standard deviation $\sigma_{j}>0$. Specifically, with probability $p_{j}$, $X_{ij}$ is considered missing and requires imputation; with probability $1 - p_{j}$, $X_{ij}$ is sampled from the non-missing abundance distribution of taxon $j$, indicating that imputation is unnecessary. The mean parameter of the normal distribution is modeled as a linear function of the sample covariates, $D_{i}^{\top}\gamma_{j}$ where $\gamma_{j}\in\mathbb{R}^{q}$ is a parameter vector that describes the impact of the $q$ covariates on the abundance of taxonomic unit $j$ (including the intercept). This modeling approach ensures that samples with similar covariates exhibit similar expected abundance values for taxon $j$, particularly when there are no missing values. The underlying intuition of the model is that the non-missing abundance of taxon $j$ in the samples follows a normal distribution, with the mean described by the sample covariates, representing the expected abundance. However, due to issues such as library preparation and undersampling during sequencing, false zeros or low counts may be introduced into the data, resulting in an additional mode close to zero in the abundance distribution of taxon $j$. To capture the mode near zero, a gamma distribution was employed.

Next, the expectation-maximization (EM) algorithm was used to fit the mixture model in order to estimate the maximum likelihood estimates of the abundance for taxa $j$, including $\hat{p}_{j}$, $\hat{\alpha}_{j}$, $\hat{\beta}_{j}$, $\hat{\gamma}_{j}$, and $\hat{\sigma}_{j}^{2}$. For certain taxon, the fitted mixture model effectively captured the bimodality of their abundance distributions. However, for taxa with unimodal abundance distributions, the normal distribution typically provided a better fit. In such cases, the EM algorithm may encounter convergence issues. To address this, the Likelihood Ratio Test (LRT) was introduced to assess whether the gamma-normal mixture model significantly outperforms the normal distribution model, $X_{ij}\mathcal{\sim N(}D_{i}^{\top}\eta_{j},\omega_{j}^{2})$, in fitting the abundance of taxon $j$. Based on the maximum likelihood estimates $\hat{\eta}_{j}$ and $\hat{\omega}_{j}^{2}$, and assuming all $X_{ij}$ are independent, the LRT statistic for taxon $j$ can be expressed as:

$\Lambda_{j}=-2\ln\frac{\prod_{i=1}^{n} f_{\mathcal{N}}\left( X_{ij};D_{i}^{\top}\hat{\eta}_{j},\hat{\omega}_{j}^{2} \right)}{\prod_{i=1}^{n} \left[ \hat{p}_{j}\cdot f_{\Gamma}\left( X_{ij};\hat{\alpha}_{j},\hat{\beta}_{j} \right)+(1-\hat{p}_{j})\cdot f_{\mathcal{N}}\left( X_{ij};D_{i}^{\top}\hat{\gamma}_{j},\hat{\sigma}_{j}^{2} \right) \right]}$ (2)

Under the null hypothesis, which assumes the normal distribution model as the true model, the statistic asymptotically follows a chi-squared distribution with 3 degrees of freedom. This is due to the three additional parameters introduced by the mixture model compared to the normal distribution model.

According to the study in the literature mbImpute, the researchers computed the LRT $p$-values on six real WGS datasets and found that only a few taxa had $p$-values exceeding 0.05. When the LRT $p$-value $\leq0.05$, the mixture model was employed to determine which taxa required imputation. Specifically, the posterior probability estimate of $X_{ij}$ being from the gamma component was computed to decide whether imputation was necessary for $X_{ij}$:

$d_{ij}=\frac{\hat{p}_{j}\cdot f_{\Gamma}\left( X_{ij};\hat{\alpha}_{j},\hat{\beta}_{j} \right)}{\hat{p}_{j}\cdot f_{\Gamma}\left( X_{ij};\hat{\alpha}_{j},\hat{\beta}_{j} \right)+(1-\hat{p}_{j})\cdot f_{\mathcal{N}}\left( X_{ij};D_{i}^{\top}\hat{\gamma}_{j},\hat{\sigma}_{j}^{2} \right)}$ (3)

Here,$f_{\Gamma}(\cdot;\hat{\alpha}_{j},\hat{\beta}_{j})$ and $f_{\mathcal{N}}(\cdot;D_{i}^{\top}\hat{\gamma}_{j},\hat{\sigma}_{j}^{2})$ represent the estimated probability density functions of the gamma and normal distributions in the mixture model, respectively. If the LRT $p$-value $>0.05$, it is concluded that the abundance of taxon $j$ does not require imputation. Based on the value of $d_{ij}$, the set $\boldsymbol{\Omega}$ is constructed. The set $\boldsymbol{\Omega}$ is defined as:

$\Omega=\{(i,j):d_{ij}<d_{\text{thre}},i=1,\ldots,n;j=1,\ldots,m\}$ (4)

The parameter $\delta$ serves as a threshold for $d_{ij}$ to determine potential missing values, with smaller values indicating lower probability of missingness. δ was set to a default value of 0.5.

Finally, to make the data in Scheme 1, three different filtering methods were designed to select taxon. Specifically, for each taxon, it was checked whether at least 40%, 50%, or 60% of the samples had non-zero counts. This filtering step aimed to remove taxon with overly sparse abundance distributions or those lacking practical significance. Taxon meeting the criteria were retained in the dataset, while those failing to meet the conditions were excluded.

After this filtering process, the following results were obtained: For the CA dataset, 80 taxa met the criteria at 40%, 115 at 50%, and 152 at 60%; for the CRC dataset, 66 taxa met the criteria at 40%, 101 at 50%, and 141 at 60%.

**Generation of complete data for Scheme 2**

To generate the complete data for Scheme 2, we first processed the raw data according to the preprocessing and normalization steps described in the Methods section. The processed abundance matrix is denoted as $\boldsymbol{X}=(X_{ij})\in\mathbb{R}^{n\times m}$, where $n$represents the number of samples and $m$ represents the number of taxa. Based on this, a set $\Omega$ was identified, similar to Scheme 1.

Subsequently, for each taxon, it was checked whether at least 85% of the samples had non-zero counts. If this condition was met, the taxon was retained; otherwise, it was filtered out from the data. The index set of taxon $j$ with non-zero counts in the samples was represented as:

$\mathcal{I}_{j}=\{i:X_{ij}>0,i=1,\ldots,n\}$ (5)

where $|\mathcal{I}_{j}|\geq n*85\%$. A new sample index set $\mathcal{I}_{j}^{'}$ was then constructed, which was a random subset of $\mathcal{I}_{j}$ with a size of 35.

We randomly ordered the 35 samples and constructed the complete data. According to the construction, the complete data contains at least 85% non-zero values. After this filtering process, the following results were obtained: For the CA dataset, 422 taxa met the criteria, while for the CRC dataset, 434 taxa were retained.

**Dropout step**

In order to mirror real situations more accurately, non-biological zeros are introduced into the data by imitating the patterns of zero values that can be seen in real data. Firstly, the mean values of all taxa within the complete dataset are calculated. According to these mean values, the masking probability for each taxon is then determined. Taxa with higher mean counts are allocated lower masking probabilities, whereas taxa with lower mean counts are assigned higher masking probabilities. This method is consistent with the actual observation that taxa having smaller counts tend to be recorded as zeros during the sequencing process. Eventually, these masking probabilities, which act as the parameters for the binomial distribution, are utilized to randomly remove values for each taxon. That is to say, if a count unit is assigned a zero by the binomial distribution, its value will be set to zero; otherwise, it will keep its original value.

**Classification**

The classification results are evaluated through the application of 5-fold cross-validated precision recall area under the curve (PR-AUC). Specifically, the DA taxa determined by difference abundance analysis are taken as the feature, the binary classification of disease/health is performed using R package glmnet (version 4.1.8),

and the PR-AUC is computed using R package proroc (version 1.3.1).

**Supplementary Figures**

Fig. S1: Box plots of MSE to evaluate mbSparse’s performance with datasets containing varying percentages numbers of outlier samples, across different sequencing depths and based on thirty replicates of simulated datasets. Imputed results are used as the baseline.

Fig. S2: The histogram showing the p-value distribution from differential abundance analysis performed using LEfSe on the raw datasets and imputed datasets from *Feng et al*., *Hale et al.*, and *Wirbel et al..*

Fig. S3: The histogram showing the p-value distribution from differential analysis performed using Wilcoxon on the raw datasets and imputed datasets from *Feng et al*., *Hale et al.*, and *Wirbel et al..*

Fig. S4: The histogram showing the p-value distribution from differential analysis performed using DESeq2-phyloseq on the raw datasets and imputed datasets from *Feng et al*., *Hale et al.*, and *Wirbel et al..*

Fig. S5: The histogram showing the p-value distribution from differential analysis performed using edgeR on the raw datasets and imputed datasets from *Feng et al*., *Hale et al.*, and *Wirbel et al..*

Fig. S6: The histogram showing the p-value distribution from differential abundance analysis performed using ALDEx2 on the raw datasets and imputed datasets from *Feng et al*., *Hale et al.*, and *Wirbel et al..*

Fig. S7: The scatter plots illustrate the relationships between the abundances of microbial pairs, including microbial pairs that are significantly altered in individuals with CRC such as *Alistipes finegoldii*-*Gemella haemolysans* and *Faecalibacterium prausnitzii*-*Bifidobacterium dentium*, as well as randomly selected microbial pairs like *Anaerostipes hadrus*-*Rothia mucilaginosa* and *Bacteroides stercoris*-*Gemella haemolysans*. The analysis compares data based on sample status, with and without the use of mbSparse as a preprocessing step. The left panels show two SMA regression lines based on the non-imputed data and their corresponding Pearson correlations (black: based on all samples; blue: based on samples where both taxa have non-zero abundances). The right panels display the SMA regression line (blue) and Pearson correlation using all samples from the mbSparse-imputed data.

Fig. S8: The bar chart showing the precision, recall, and F1 scores of LEfSe DA methods on both non-imputed data and data imputed by mbSparse in the 16S data simulation.

Fig. S9: The bar chart showing the precision, recall, and F1 scores of Wilcoxon DA methods on both non-imputed data and data imputed by mbSparse in the 16S data simulation.

Fig. S10: The bar chart showing the precision, recall, and F1 scores of DESeq2-phyloseq DA methods on both non-imputed data and data imputed by mbSparse in the 16S data simulation.

Fig. S11: The bar chart showing the precision, recall, and F1 scores of edgeR DA methods on both non-imputed data and data imputed by mbSparse in the 16S data simulation.

Fig. S12: The bar chart showing the precision, recall, and F1 scores of ALDEx2 DA methods on both non-imputed data and data imputed by mbSparse in the 16S data simulation.

**Supplementary Tables**

Table S1: The 2x2 contingency table gives the confusion matrix for DA taxa identified through edgeR analysis on the non-imputed data. The rows and columns indicate whether or not the taxa were annotated with a CRC functional term, respectively. Each entry in the table represents the number of taxa that meet the conditions specified by the corresponding rows and columns. The datasets used for this analysis include those from the CRC WGS datasets of Feng et al., Hale et al., and Wirbel et al.

|  | **Identified as DA** | **Not identified as DA** |
| --- | --- | --- |
| Annotated by the term | 57 | 76 |
| Not annotated by the term | 723 | 808 |

Table S2: The 2x2 contingency table gives the confusion matrix for DA taxa identified through edgeR analysis on the mbSparse-imputed data. The rows and columns indicate whether or not the taxa were annotated with a CRC functional term, respectively. Each entry in the table represents the number of taxa that meet the conditions specified by the corresponding rows and columns. The datasets used for this analysis include those from the CRC WGS datasets of Feng et al., Hale et al., and Wirbel et al.

|  | **Identified as DA** | **Not identified as DA** |
| --- | --- | --- |
| Annotated by the term | 96 | 37 |
| Not annotated by the term | 725 | 806 |

Table S3: Evaluation of mbSparse in identifying zeros due to downsampling of Feng et al.'s CRC WGS dataset. For each of two removal rates 30% and 40%, we repeat independent downsampling for thirty times.

| **Removal rate** | **30%** | **40%** |
| --- | --- | --- |
| % of downsampling zeros identified | 87.24%±1.25% | 87.28%±1.41% |
| Pearson correlation non-imputed | 0.4730±2.5178e-05 | 0.4717±0.0001 |
| Pearson correlation mbSparse-imputed | 0.8541±0.0034 | 0.8120±0.0029 |
| Spearman correlation non-imputed | 0.8547±0.0002 | 0.7972±0.0021 |
| Spearman correlation mbSparse-imputed | 0.8688±0.0064 | 0.8359±0.0093 |

Table 4: Evaluation of mbSparse in identifying zeros due to downsampling of Feng et al.'s CRC WGS dataset. For each of three removal rates 50%, 60%, and 70%, we repeat independent downsampling for thirty times.

| **Removal rate** | **50%** | **60%** | **70%** |
| --- | --- | --- | --- |
| % of downsampling zeros identified | 90.92%±1.39% | 96.12±0.84% | 99.76%±0.21% |
| Pearson correlation non-imputed | 0.4687±0.0002 | 0.4621±0.0002 | 0.4478±0.0002 |
| Pearson correlation mbSparse-imputed | 0.7719±0.0025 | 0.7317±0.0025 | 0.6781±0.0036 |
| Spearman correlation non-imputed | 0.7337±0.0002 | 0.6593±0.0003 | 0.5739±0.0029 |
| Spearman correlation mbSparse-imputed | 0.8024±0.0091 | 0.7524±0.0123 | 0.6780±0.0137 |
