# Supplementary figures and images for: mbSparse: an autoencoder-based imputation method to address sparsity in microbiome data
Source: Gut Microbes. 2025 Sep 1;17(1):2552347. doi: 10.1080/19490976.2025.2552347 (PMC12407639; doi:10.1080/19490976.2025.2552347)

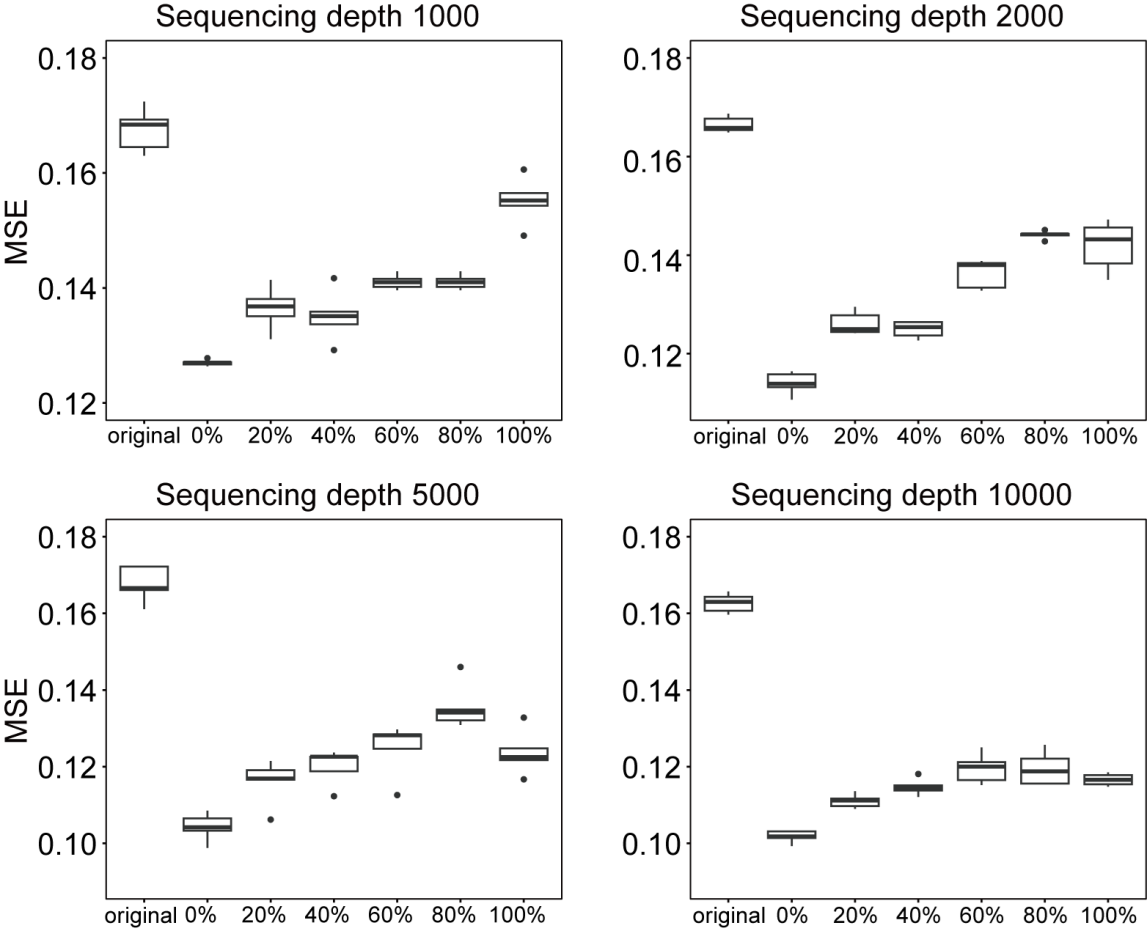

Supplement: Supplementary_data.zip [file KGMI_A_2552347_SM8816.zip › Supplementary Figures/Figure S1.tif]

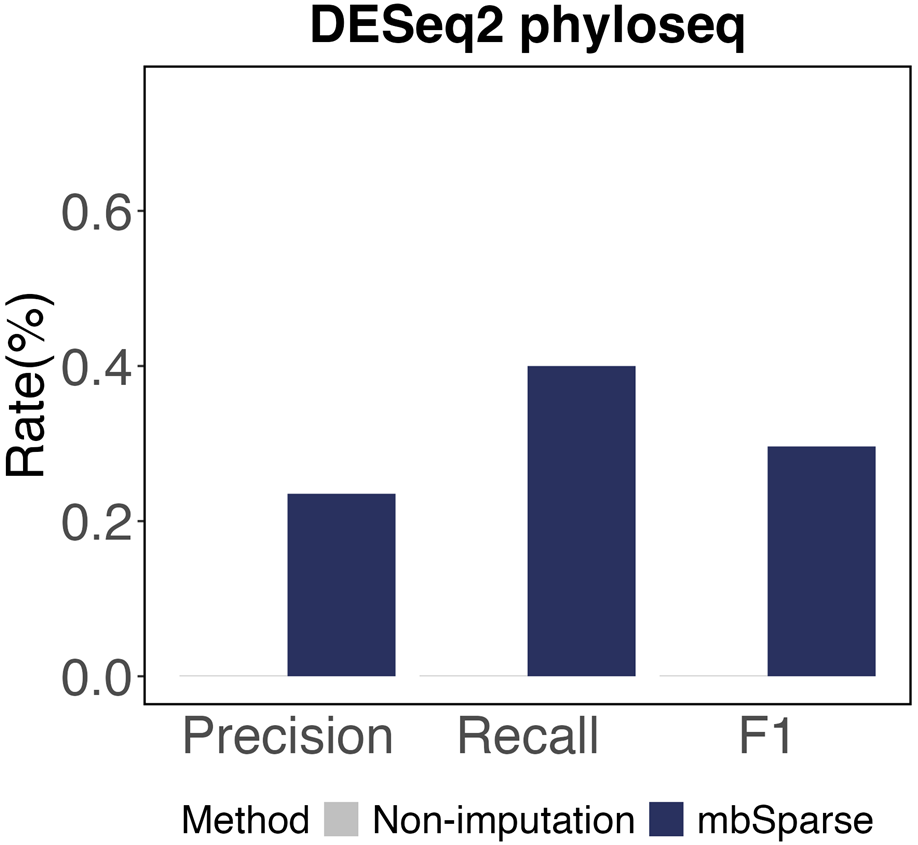

Supplement: Supplementary_data.zip [file KGMI_A_2552347_SM8816.zip › Supplementary Figures/Figure S10.tif]

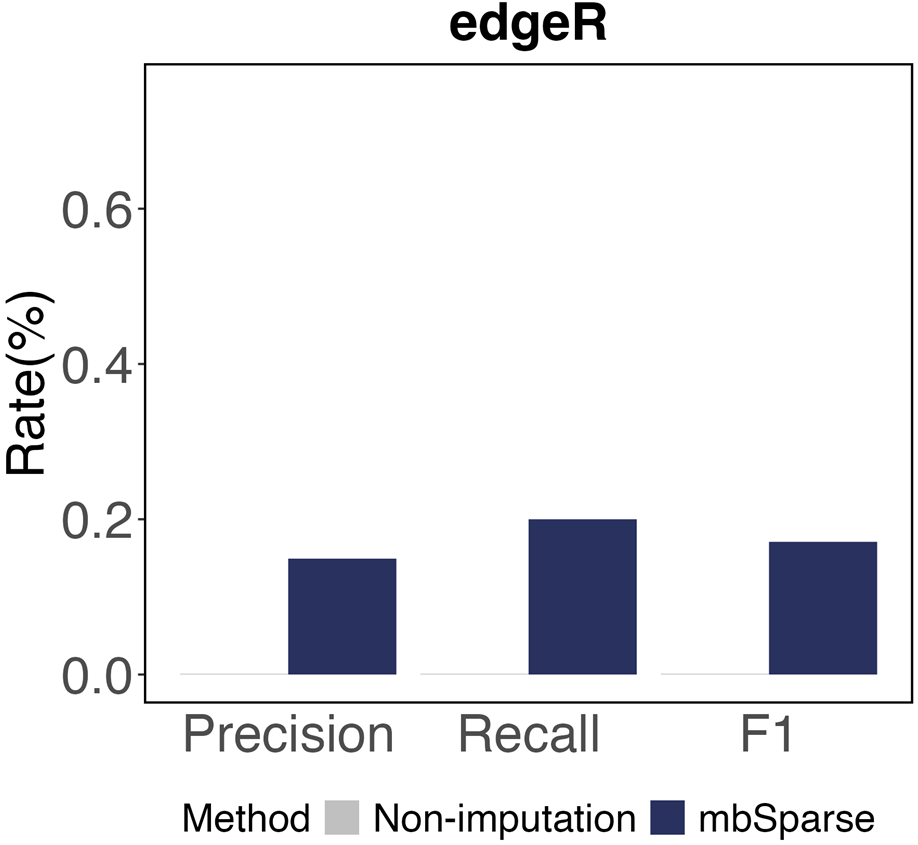

Supplement: Supplementary_data.zip [file KGMI_A_2552347_SM8816.zip › Supplementary Figures/Figure S11.tif]

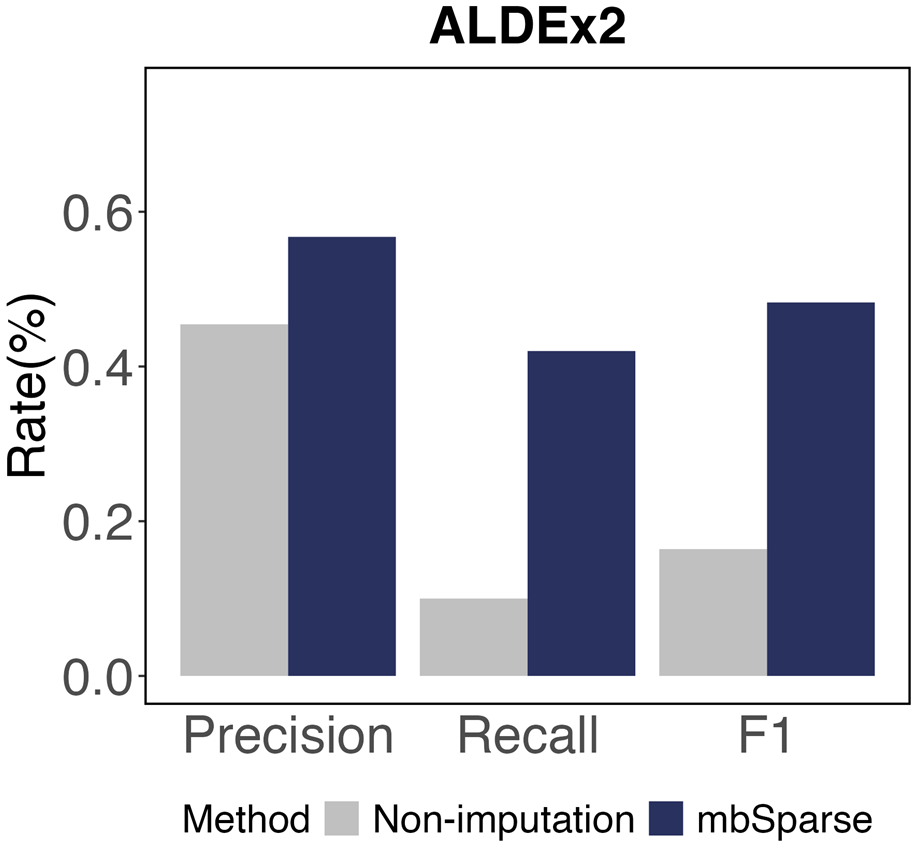

Supplement: Supplementary_data.zip [file KGMI_A_2552347_SM8816.zip › Supplementary Figures/Figure S12.tif]

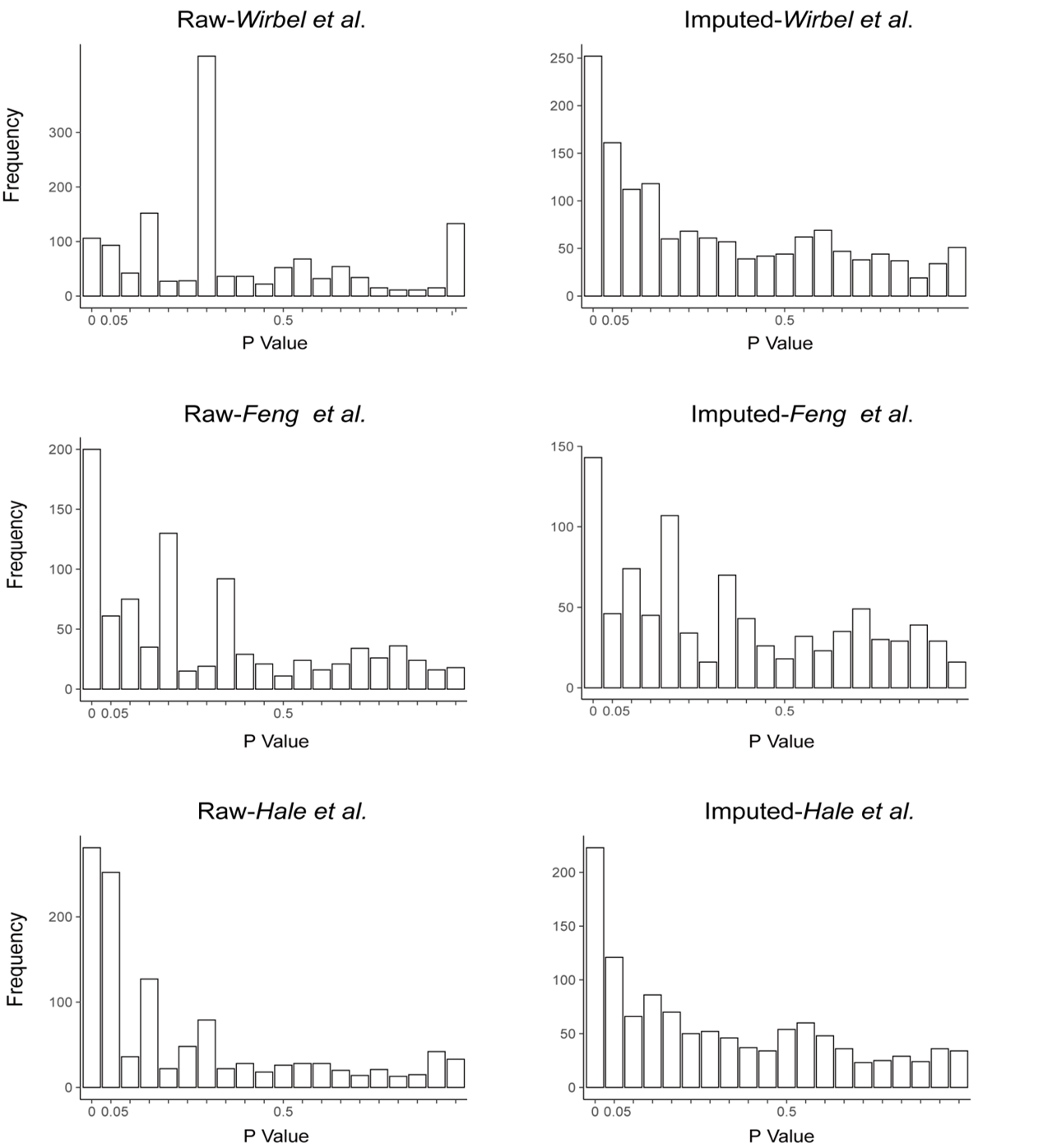

Supplement: Supplementary_data.zip [file KGMI_A_2552347_SM8816.zip › Supplementary Figures/Figure S2.tif]

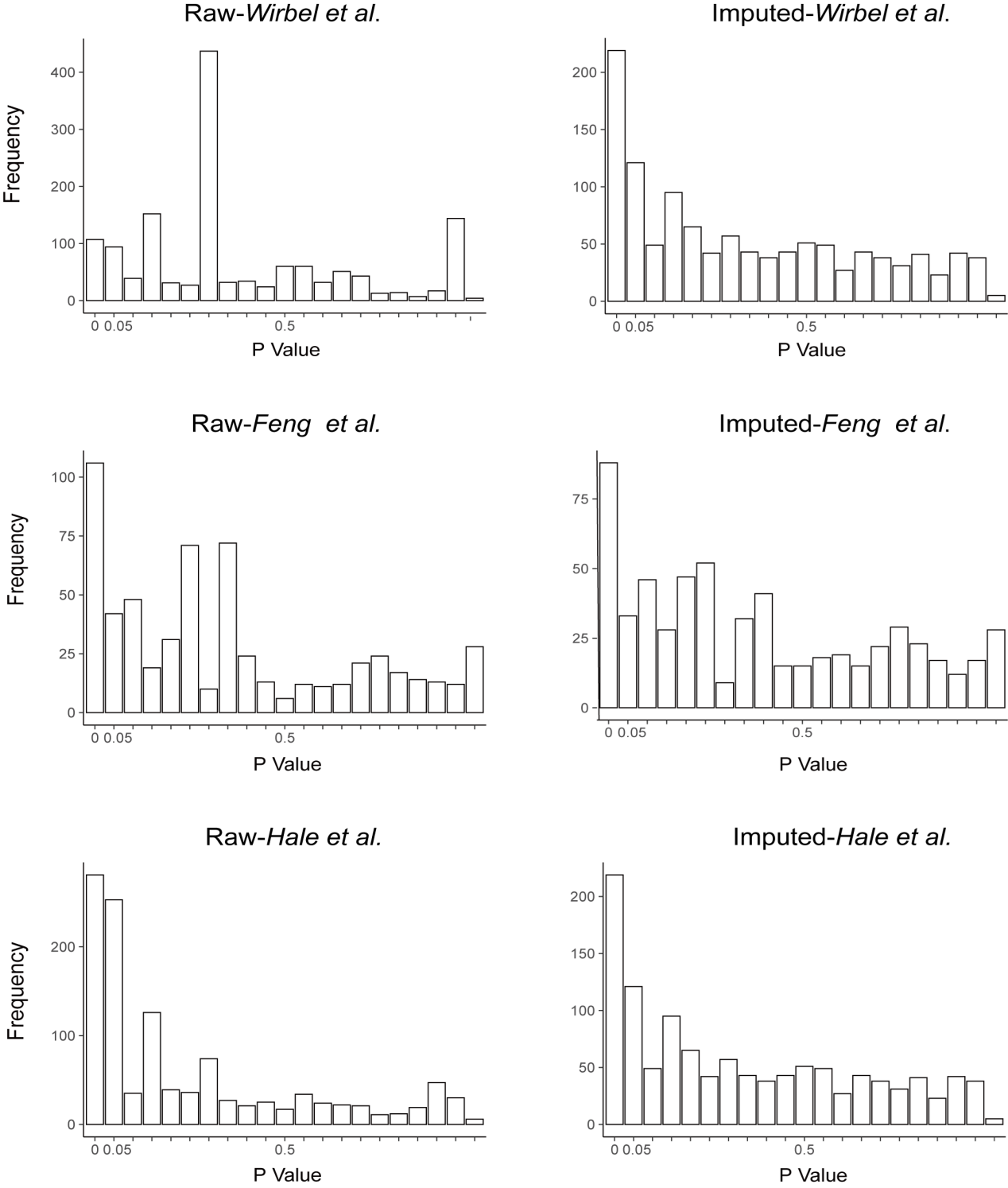

Supplement: Supplementary_data.zip [file KGMI_A_2552347_SM8816.zip › Supplementary Figures/Figure S3.tif]

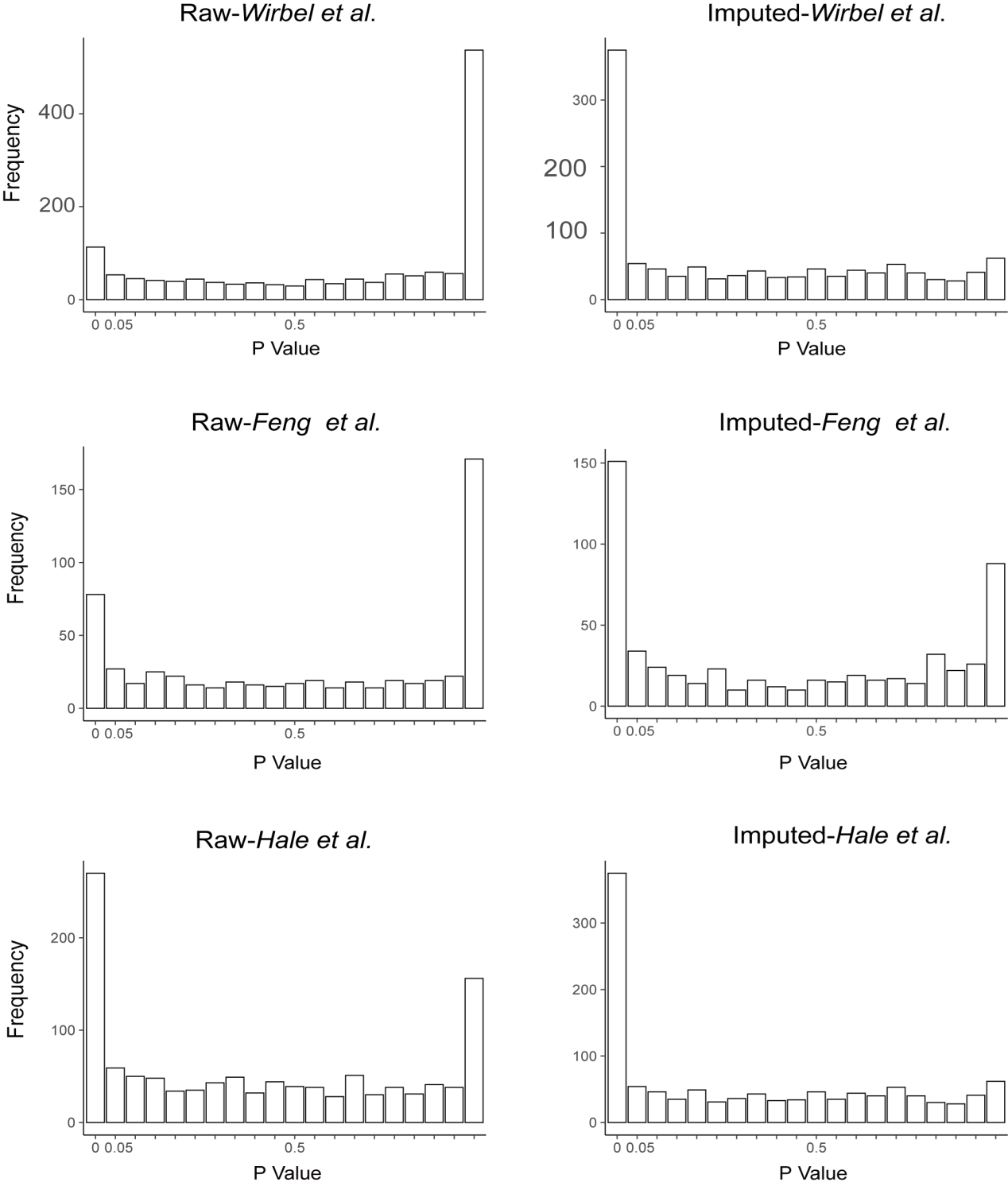

Supplement: Supplementary_data.zip [file KGMI_A_2552347_SM8816.zip › Supplementary Figures/Figure S4.tif]

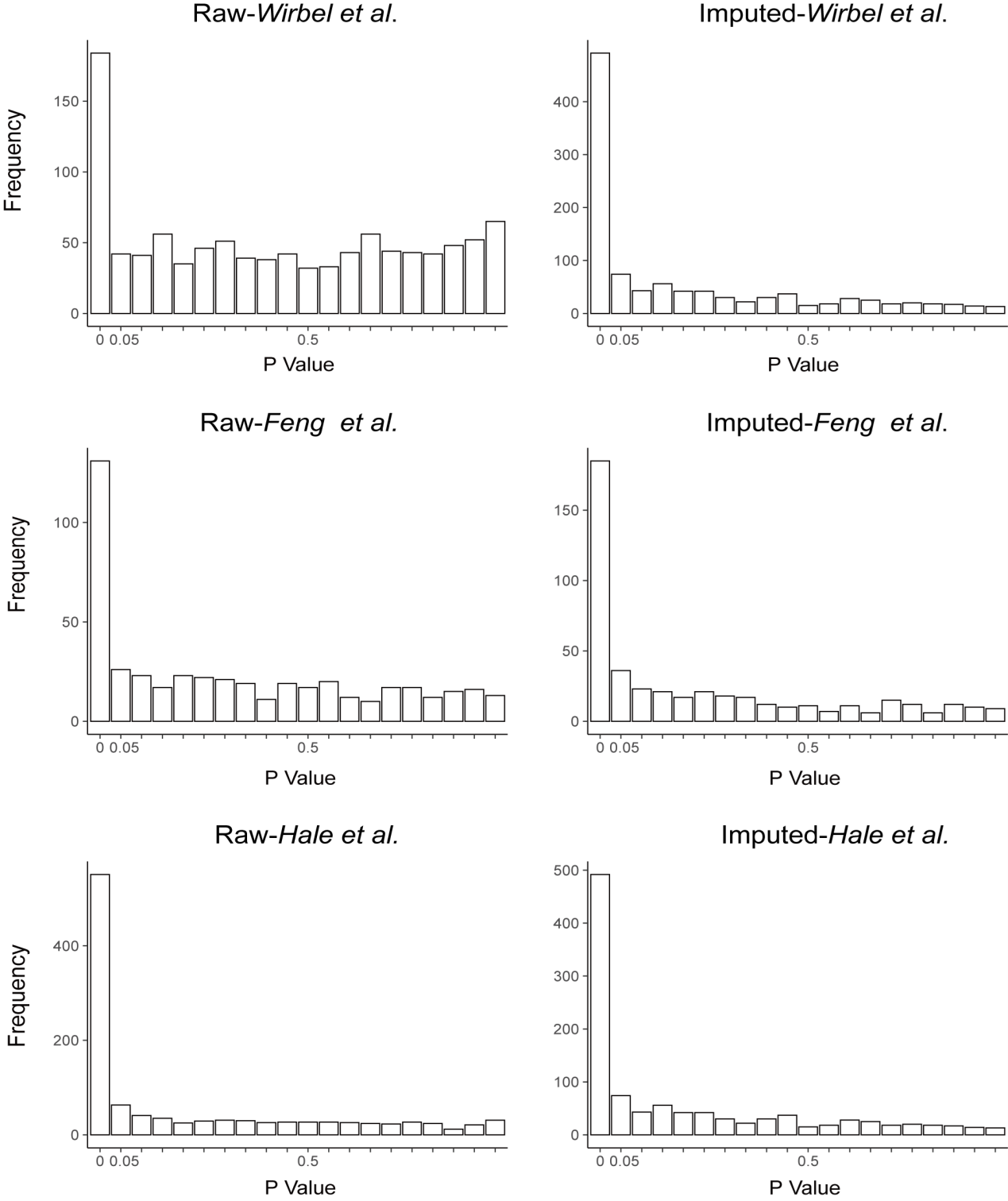

Supplement: Supplementary_data.zip [file KGMI_A_2552347_SM8816.zip › Supplementary Figures/Figure S5.tif]

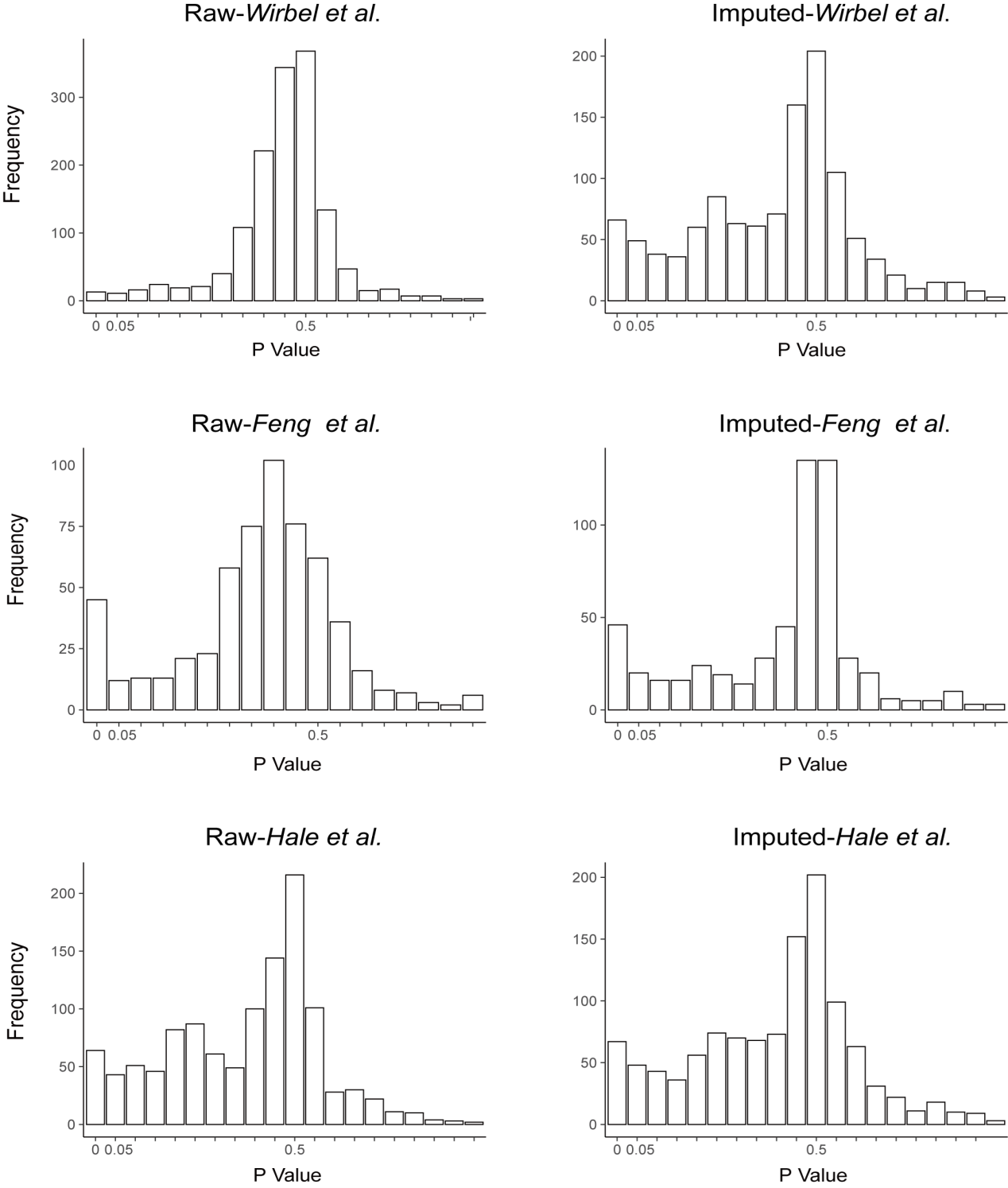

Supplement: Supplementary_data.zip [file KGMI_A_2552347_SM8816.zip › Supplementary Figures/Figure S6.tif]

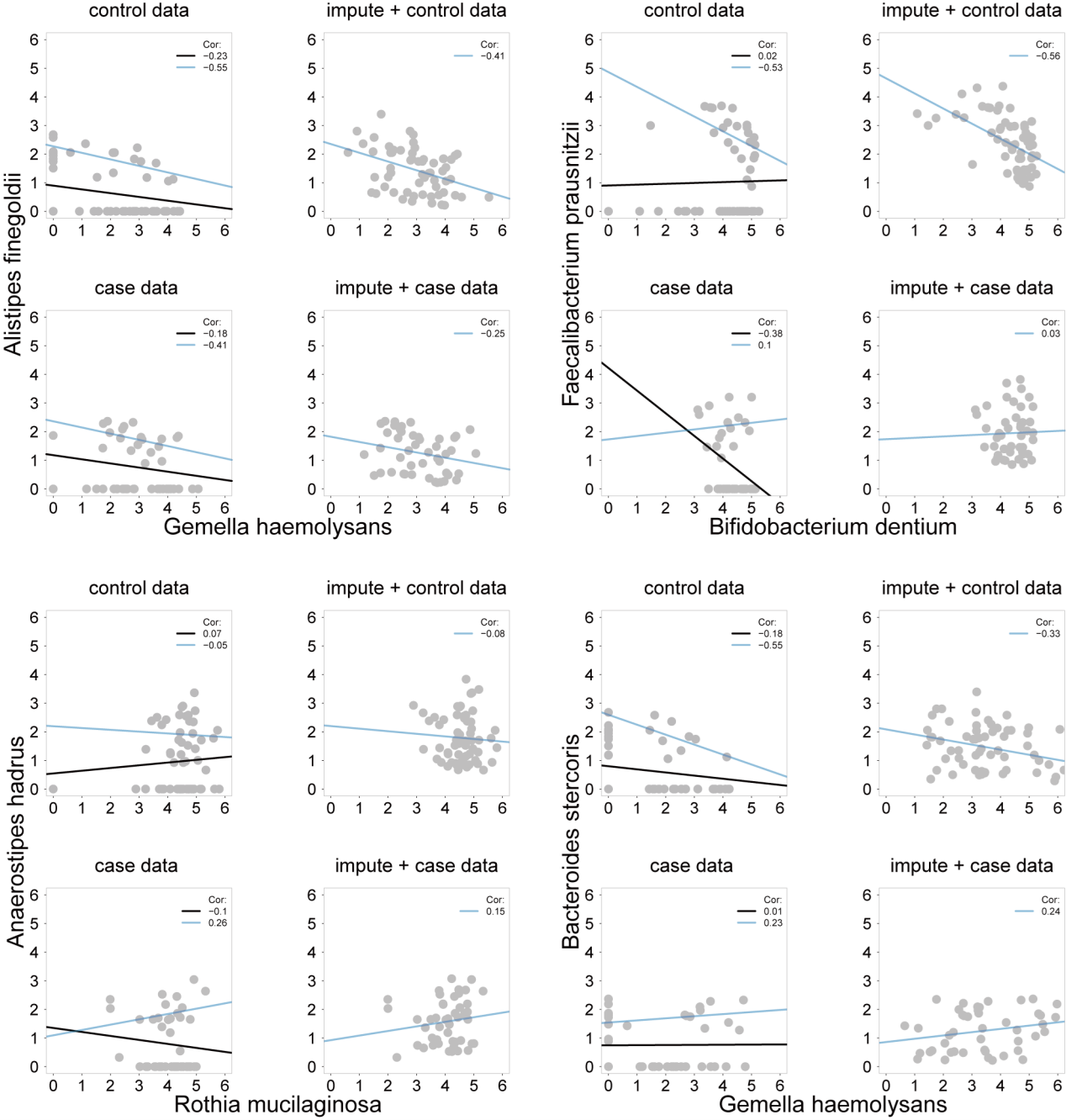

Supplement: Supplementary_data.zip [file KGMI_A_2552347_SM8816.zip › Supplementary Figures/Figure S7.tif]

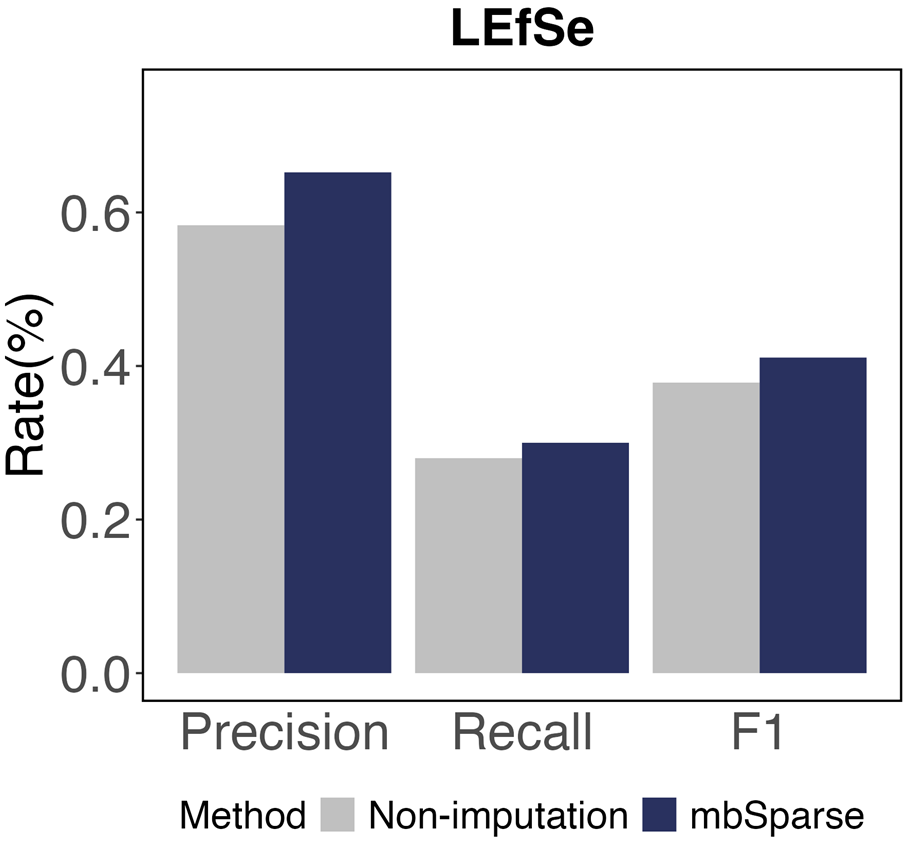

Supplement: Supplementary_data.zip [file KGMI_A_2552347_SM8816.zip › Supplementary Figures/Figure S8.tif]

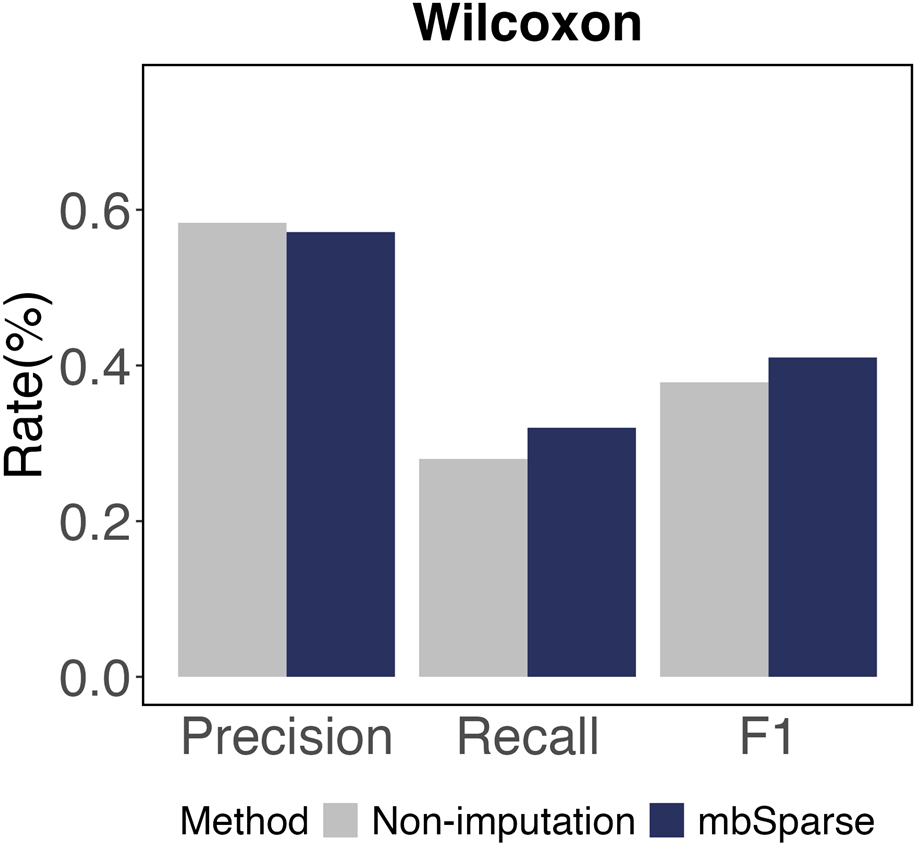

Supplement: Supplementary_data.zip [file KGMI_A_2552347_SM8816.zip › Supplementary Figures/Figure S9.tif]
